# *Caenorhabditis elegans* Infrared-Based Motility Assay Identified New Hits for Nematicide Drug Development

**DOI:** 10.3390/vetsci6010029

**Published:** 2019-03-17

**Authors:** Gastón Risi, Elena Aguilera, Enrique Ladós, Gonzalo Suárez, Inés Carrera, Guzmán Álvarez, Gustavo Salinas

**Affiliations:** 1 Worm Biology Laboratory, Institut Pasteur de Montevideo, Montevideo 11400, Uruguay; gastonrisi@gmail.com (G.R.); quiquelados@gmail.com (E.L.); inescarrera@fq.edu.uy (I.C.); 2 Grupo de Química Medicinal, Facultad de Ciencias, Universidad de la República, Montevideo 11400, Uruguay; eaguilera@fcien.edu.uy; 3 Área Farmacología, Departamento de Fisiología, Facultad de Veterinaria, Universidad de la República, Montevideo 11600, Uruguay; suarezveirano@gmail.com; 4 Departamento de Ciencias Farmacéuticas, Área Farmacología, Facultad de Química, Universidad de la República, Montevideo 11800, Uruguay; 5 Laboratorio de Moléculas Bioactivas—CENUR Litoral Norte, Universidad de la República, Paysandú 60000, Uruguay; 6 Departamento de Biociencias, Facultad de Química, Universidad de la República, Montevideo 11400, Uruguay

**Keywords:** nematicide, nematode, anthelmintic, *C. elegans*, arylidene ketones, aryl hydrazine, “Pathogen Box”

## Abstract

Nematode parasites have a profound impact on humankind, infecting nearly one-quarter of the world’s population, as well as livestock. There is a pressing need for discovering nematicides due to the spread of resistance to currently used drugs. The free-living nematode *Caenorhabditis elegans* is a formidable experimentally tractable model organism that offers key advantages in accelerating nematicide discovery. We report the screening of drug-like libraries using an overnight high-throughput *C. elegans* assay, based on an automated infrared motility reader. As a proof of concept, we screened the “Pathogen Box” library, and identical results to a previous screen using *Haemonchus contortus* were obtained. We then screened an in-house library containing a diversity of compound families. Most active compounds had a conjugation of an unsaturation with an electronegative atom (N, O, or S) and an aromatic ring. Importantly, we identified symmetric arylidene ketones and aryl hydrazine derivatives as novel nematicides. Furthermore, one of these compounds, (1E,2E)-1,2-bis(thiophen-3-ylmethylene)hydrazine, was active as a nematicide at 25 µm, but innocuous to the vertebrate model zebrafish at 50 µm. Our results identified novel nematicidal scaffolds and illustrate the value of *C. elegans* in accelerating nematicide discovery using a nonlabor-intensive automated assay that provides a simple overnight readout.

## 1. Introduction

Nematoda (commonly known as roundworms) is one of the largest phyla in the animal kingdom and includes free-living as well as parasitic species. Nematode parasites have a profound impact on humankind. Approximately one-quarter of the world’s population harbors nematode infections, with the greatest prevalence in Africa, Asia, and Latin America [1,2,3]. Nematodes also infect livestock and crops, affecting food production [1]. Resource-poor regions of the world suffer under an enormous burden of ill health, adverse social impacts, and economic loss caused by nematode infections [4].

Nematodes are the most common human parasites. Nearly 1 billion people are infected with *Ascaris lumbricoides*, the major cause of human geohelminthiasis, while several hundred million people are infected with other soil-transmitted nematodes, mainly whipworms such as *Trichuris trichiura* and hookworms such as *Necator americanus* and *Ancylostoma duodenalis* [3]. These nematodes colonize the gut and often result in diarrhea and anemia. Tissue-dwelling nematodes are less prevalent, but can cause serious illness. Lymphatic filariasis affects 120 million people worldwide and can result in gross disfiguration and enlargement of the lower limbs. Several million people are thought to be infected with *Onchocerca volvulus*, which can cause impaired sight leading to “river blindness” [5,6]. Nematodes are also the most common parasites of livestock (notably cattle and sheep, but also pigs and horses), impacting animal welfare and causing significant economic loss [7]. *Haemonchus* spp., *Cooperia* spp., and *Ostertagia* spp. are the most prevalent parasitic worms in livestock and cause important damage. Since thousands of *H. contortus* adult worms can infect a single host and each of them can remove up to 30 µL of blood per day, infection rapidly causes anemia, edema, and consequent death. This parasite, with the shortest life cycle (20 days) of any gastrointestinal nematode, possesses an enormous biotic potential: Each female worm lays 5000 to 15,000 eggs per day [8].

**Figure 1:**
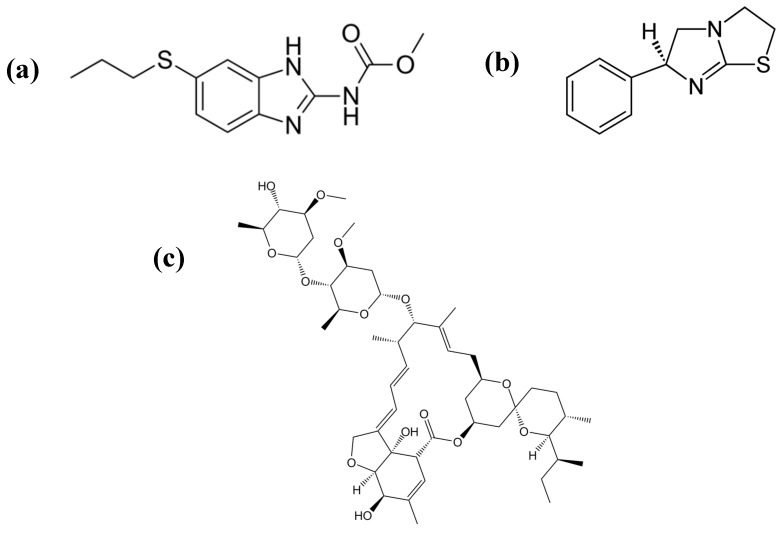
Main classes of nematicides: (**a**) Albendazole, (**b**) levamisole, and (**c**) ivermectin.

Despite the magnitude of the health and socioeconomic problems caused by worm infections, there are few solutions. The first veterinary vaccine against a nematode (*H. contortus*) has recently been developed and commercially launched (BarberVax^®^), and is currently under field trial evaluation. The control of nematode infections has traditionally relied on chemotherapy. In livestock, there is widespread resistance to most classes of nematicides (e.g., benzimidazoles (albendazole), avermectins (ivermectin), imidazothiazoles (levamisole)) due to their indiscriminate use [2,9,10,11,12] (Figure 1).

The emergence and spread of drug resistance is a threat to human nematode infections. Therefore, there is a pressing need for new nematicides. The shortage of new drugs is due to several reasons, among them the extraordinary success of ivermectin, an anthelmintic to which there is now widely reported resistance in livestock [10,13]. More fundamentally, research on parasitic worms has received much less funding than other globally important health issues [14]. There is low funding for research in poor countries, and pharmaceutical industries do not allocate resources due to the low profitability of investments in this field [4]. 

The lack of a convenient model to perform high-throughput screening (HTS) of chemical libraries is another obstacle for drug discovery, because parasitic worms cannot be well-cultured without a host, and phenotypic drug screens are labor-intensive. 

The model organism *Caenorhabditis elegans* is a free-living nematode that offers a great opportunity for use as a “model parasite” in accelerating drug discovery against nematodes [15,16,17]. The experimental tractability of *C. elegans* and the solid scientific community around this model organism are some of the key advantages of this model. Although *C. elegans* is not a parasite, it is closely related to parasitic nematodes [18]. Despite dissimilar lifestyles of free-living and parasitic species and genetic differences associated with parasitism (specific gene losses and gene family expansions associated with parasitism), *C. elegans* is as similar to parasitic worms as parasitic worms are to each other [19]. Significantly, most nematicides kill *C. elegans*. Indeed, *C. elegans* has been instrumental in elucidating the mechanism of nematicidal activity and resistance [20]. The molecular targets for the drugs that come out of screens can be defined using the powerful approach of forward genetics in *C. elegans* and may uncover new effectors for nematicidal activity. Recently, *C. elegans* was used in a screen of 67.012 compounds, and the results were encouraging: More than half of the lethal compounds against *C. elegans* were also lethal against *H. contortus* and/or *Cooperia* spp. In addition, a small number of them were lethal to *Danio rerio*, demonstrating the selectivity of this process [15]. However, this screening was time-consuming and required visualization and image processing. 

Different *C. elegans* assays have been developed for nematicide discovery, particularly using behavioral and metabolic readouts. Colorimetric and fluorescence-based assays have been reported for metabolism analysis [21,22]. Regarding behavior, the range of approaches is broader. The usage of a scanner to identify decreased motility has been reported [23]. There are different automatic systems for assessing and analyzing behavioral changes, including worm tracking by beam interruption or camera systems and microfluidics devices, among others [24].

Progress in drug discovery also relies on high-quality chemical libraries that can be used for HTS as a basis for subsequent research. These libraries do not abound. One such curated library is “Pathogen Box”, which contains 400 diverse drug-like compounds and is provided freely to research labs to boost drug discovery, share results, and perform cross-laboratory validation of hits. Using this library, a single compound, tolfenpyrad, was recently found to be active against the nematode parasite *H. contortus* using a laborious manual screen [25]. 

In the present study, we validated an overnight standardized and automated HTS motility assay using the free-living nematode *C. elegans*, screened the Pathogen Box library plus 175 compounds from our in-house library (LIDENSA Chemolibrary), and identified novel scaffolds for nematicide drug development. 

## 2. Materials and Methods

### 2.1. Chemicals

Levamisole and ivermectin were obtained from Compañía Cibeles S.A. (Montevideo, Uruguay). Pathogen Box was kindly provided by Medicines for Malaria Venture: Pathogen Box plates were maintained at −20 °C until use. The second compound library (LIDENSA Chemolibrary) utilized in this study was previously synthesized as part of our ongoing program in drug development for Chagas disease and other human maladies. This library contains, to date, more than 2000 compounds [26,27,28,29,30]. We selected 60 compounds with antiparasitic activity (against *Trypanosoma cruzi*, *Trypanosoma brucei*, *Leishmania* spp., and *Fasciola hepatica*), as well as compounds randomly selected, representing all the chemical families present in our chemolibrary. The random selection of the other 115 compounds was performed based on several criteria: abundance of the compound, solubility, cost-effective synthetic procedures, and toxicology data. The structural details of the selected molecules are in Supplementary Materials Table S1. All other reagents used in this work were from SIGMA Chemical Company.

### 2.2. C. elegans, Strains, and Culture Methods

A wild-type *C. elegans* Bristol strain N2 and *Escherichia coli* (*E. coli*) OP50 strain were obtained from the Caenorhabditis Genomics Center (Minneapolis, MN, USA). The worms were maintained under standard conditions at 20 °C on nematode growth media (NGM) agar plates seeded with *E. coli* OP50 as a source of food. The wild-type *C. elegans* Bristol strain N2 was cultured and maintained according to the procedures described previously [31].

### 2.3. Assessment of C. elegans Motility Using WMicrotracker ONE

**Figure 2:**
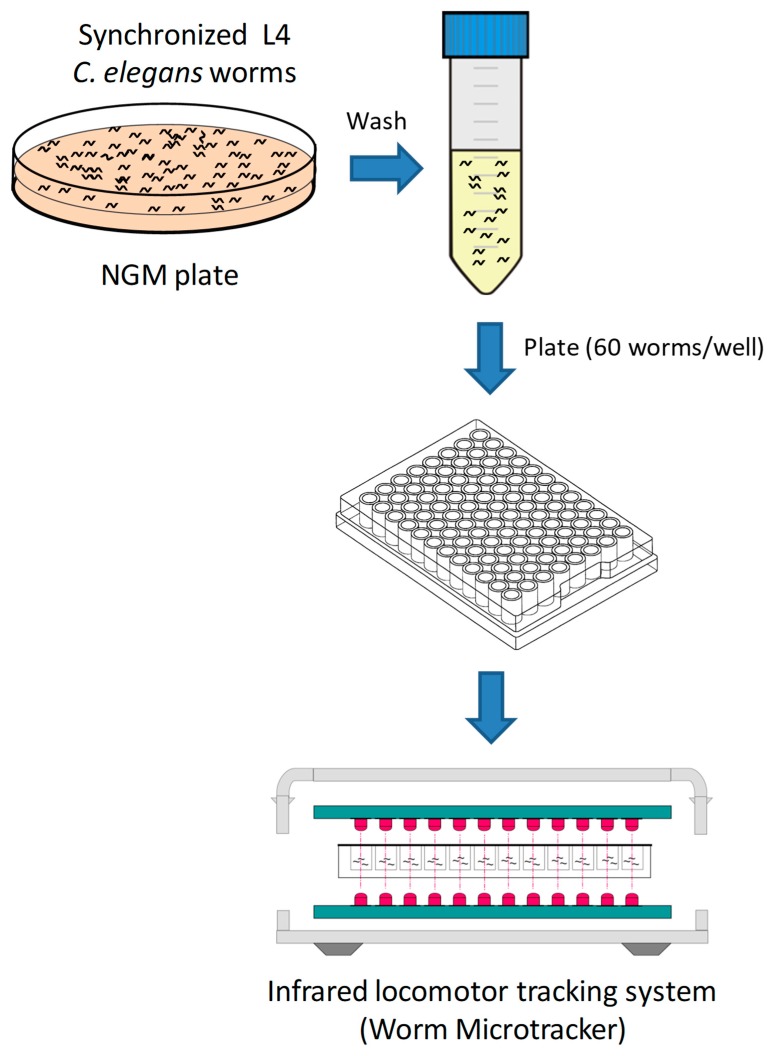
*Caenorhabditis elegans* motility screen. The motility-based screening was based on an infrared detection system (WMicrotracker^TM^ ONE, Phylumtech). An infrared system within the equipment measured *C. elegans* movement in microtiter plates. This allowed for identifying molecules that affected worm movement. The scheme of WMicrotrackerTM ONE was kindly provided by Phylumtech.

The method used to assess motility is described in detail in Reference [32]. Briefly, a locomotor activity recording system, WMicrotrackerTM ONE (PhylumTech, Santa Fe, Argentina), detects infrared microbeam interruptions (Figure 2). When worms move across the light beam, a transient fluctuation in the signal received by the phototransistor is generated, and movement is detected by digital analysis of the phototransistor output. Synchronized L4 *C. elegans* worms were removed from culture plates and washed three times with K saline (NaCl 51 mM, KCl 32 mM) by centrifugation at 1000 g. Worms were plated in 96-well flat microtiter plates (Costar). Approximately 60 worms per well were seeded in 80 µL of K saline containing 0.015% bovine serum albumin (BSA), and their basal movement was measured for 30 min to normalize the 100% movement activity for each well at the beginning of the assay. Then, the drugs were added to a final volume of 100 µL per well. A dose-dependent assay for ivermectin was carried out in the 0.01–10 µm range in 1% DMSO. The levamisole dose–response assay concentration range was 1–1000 µm. No DMSO was used in the levamisole assays. Motility, using WMicrotrackerTM ONE, was measured for 90 min.

### 2.4. Pharmacodynamic Function

The half-maximal effective concentrations (EC50) of ivermectin, levamisole, tolfenpyrad, and compound 1381 were calculated using GraphPad Prism software (GraphPad Prism, version 7.00 for Windows, San Diego, CA, USA, www.graphpad.com), fitting a dose–response sigmoid curve.

### 2.5. Screening of Compound Libraries

Following the protocol described above, Pathogen Box compounds were added at a 50-µm final concentration in 1% DMSO in a final volume of 100 µL per well. Control wells with vehicle only were assayed by octuplicate. Motility (using WMicrotracker^TM^ ONE) was measured for 17.5 h. Putative positives were rescreened using the same method. The dose-dependent motility response for tolfenpyrad was carried out in the 2–30 µm range in 1% DMSO. The compounds of the LIDENSA Chemolibrary were initially tested at 50 µm (or at a lower concentration if insoluble or limited in quantity, see Table S1). Putative positives were rescreened. The dose-dependent motility response for 1381 was carried out in the 5–50 µm range in 1% DMSO. Tolfenpyrad and 1381 toxicity at 50 µm against all developmental stages of *C. elegans* was assessed by stereoscopic microscope observation of larval and adult worms after overnight treatment at 20 °C. Lethality was determined as the absence of movement at 24 h and the absence of response to a gentle touch with a sterilized platinum wire, and was further assessed by the absence of offspring.

### 2.6. In Vivo Toxicity on Zebrafish (Danio rerio) Larvae

Two zebrafish adult males and females were placed the night before spawning in breeding tanks to let them cross, using glass marbles as a spawning substrate. Collected fertilized eggs were maintained in Petri dishes with E3 embryo medium (5 mM NaCl, 0.17 mM KCl, 0.33 mM CaCl2, 0.33 mM Mg2SO4, and 0.0001% methylene blue) at 28 °C. Seventy-two hours post fertilization, zebrafish embryos were placed in 96-well plates (3 embryos per well, 6 wells per condition) containing 225 µL of E3 medium and incubated for an additional 24 h at 28 °C. Compounds were tested at 5–50 µm concentration in a total of 250 µL/well volume. DMSO at a 1% final concentration was used to avoid compound precipitation. Viability was assessed by the presence of a heartbeat under stereoscopic microscope observation after 24 h of treatment [30].

### 2.7. Ethics Statement

The zebrafish embryos used in this work were under 120-h of postfertilization. Therefore, according to Uruguayan legislation and National Honorary Committee for Animal Experimentation (CHEA) guidelines, there was no requirement for ethical protocol approval.

## 3. Results

**Figure 3:**
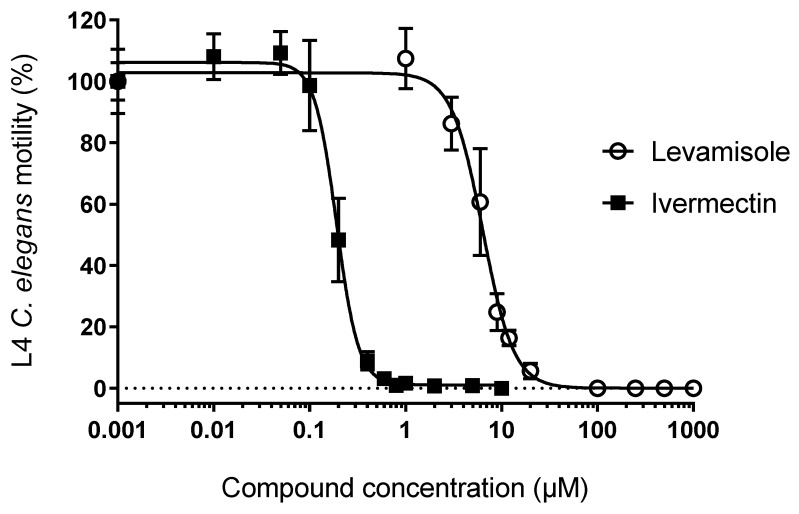
Ivermectin and levamisole effects on *C. elegans* motility: 90-min ivermectin (filled squares) and levamisole (open circles) dose–response motility assay of *C. elegans* L4 larvae. The worms were incubated in K saline containing 0.015% bovine serum albumin (BSA) and 1% DMSO. Ivermectin was examined in the 0.01–10 µm range and levamisole in the 1–1000 µm range. EC50 for ivermectin and levamisole was 0.19 ± 0.01 µm and 6.4 ± 0.3 µm, respectively. Error bars correspond to standard deviations.

Different conditions were initially tested to obtain an optimal *C. elegans* motility response. A linear correlation between worm number and motility was observed between 30 and 90 worms (data not shown). The addition of BSA was found to reduce the binding of worms to the plastic surface, and 1% DMSO (used as a compound vehicle) did not affect *C. elegans* motility. Initially, we tested two commercially available nematicides (ivermectin and levamisole) known to kill worms by paralysis through different mechanisms (reviewed in Reference [20]). The dose–response curves obtained for both compounds (Figure 3) clearly showed that the assay was suitable for screening drugs that affect motility. The EC50 was 0.19 ± 0.01 µm for ivermectin and 6.4 ± 0.3 µm for levamisole.

**Figure 4:**
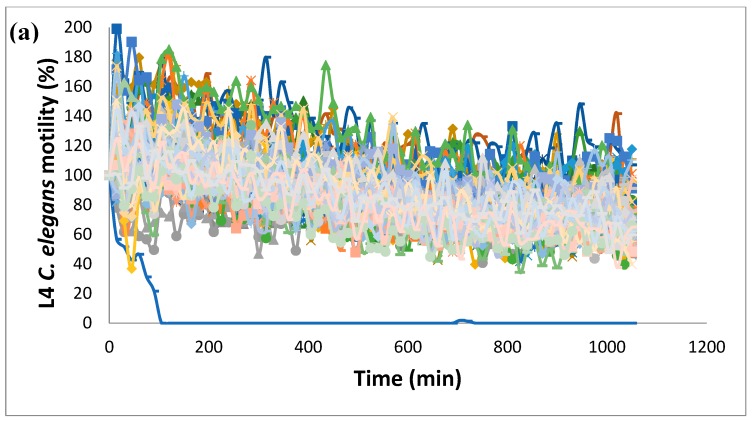

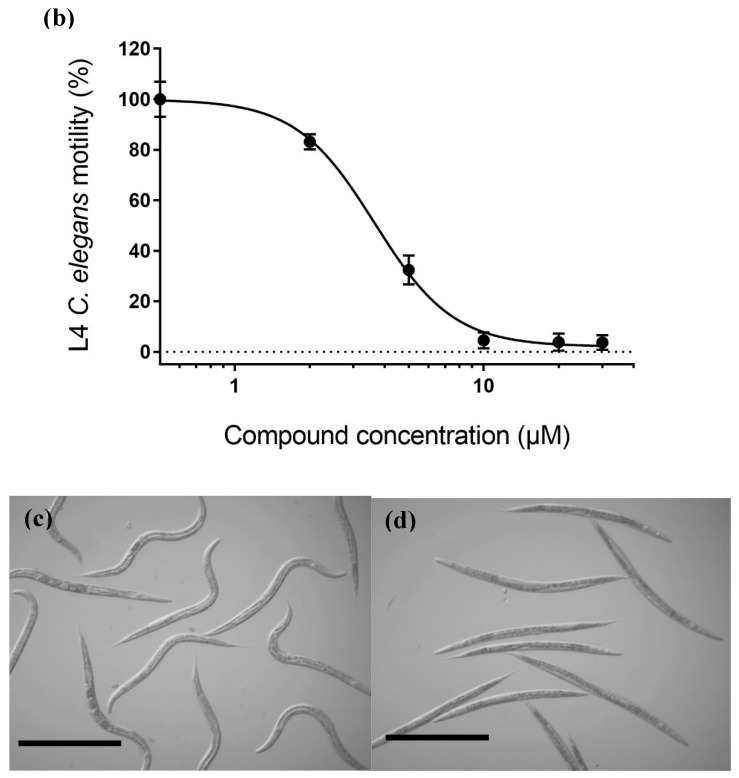
Pathogen Box screen using *C. elegans*. (**a**) Motility assay of fourth-stage larvae (L4) of *C. elegans* incubated at a 50-µm concentration of each compound in K saline containing 0.015% BSA and 1% DMSO. Each curve represents the movement of the worms in every well containing a single compound. The blue curve at the bottom is tolfenpyrad, the single hit found in the screening. A single plate is shown. (**b**) Overnight (17.5 h) tolfenpyrad dose–response motility assay of L4 *C. elegans* larvae. The worms were incubated in K saline containing 0.015% BSA, 1% DMSO, and tolfenpyrad in the 2–30 µm range. The EC50 was 3.6 ± 0.2 µm. Error bars correspond to standard deviations. Eight replicates were performed for each concentration. (**c**,**d**) Effect of tolfenpyrad on *C. elegans*. Fourth-stage larvae (L4) *C. elegans* worms were incubated with vehicle (Panel C) or with tolfenpyrad at 50 µm (Panel D) for 18 h. Scale bar: 500 µm.

Since after 20 h, *C. elegans* motility started to decrease, the endpoint of the assay was set at 17.5 h for screening purposes. As a proof of concept of the assay, we performed a screen of Pathogen Box, a library that contains 400 diverse drug-like compounds and has been previously screened for nematicides using *H. contortus*. This screen resulted in the identification of tolfenpyrad (Compound ID: MMV688934; pubchem.ncbi.nlm.nih.gov/compound/10110536) as the single active compound present in the library (Figure 4a). Tolfenpyrad was the single compound identified when this library was screened using *H. contortus* L3 and L4 larval stages. The dose–response curve showed that tolfenpyrad was fully active at a concentration as low as 10 µm (Figure 4b), with an EC50 of 3.6 ± 0.2 µm. The worms treated with tolfenpyrad were then examined under a stereoscopic microscope, and a clear lethal phenotype was observed (Figure 4c,d). Furthermore, treated L4 did not develop into adult worms, confirming the lethal phenotype.

In order to identify new nematicides, we screened 175 compounds from our in-house library (Table S1). This screening led to the identification of 28 compounds with mild to potent nematicide activity (Table 1), using an arbitrary threshold: 25%–65% remaining motility was considered mild activity, and 0%–25% remaining motility was considered potent activity. Three of the most active compounds (chemolibrary codes 795, 796, and 1245) were symmetric arylidene ketones. Two additional potent compounds were aryl hydrazine derivatives 1381 and 1140. The chalcone 731 was also identified as potent. 

**Figure 5:**
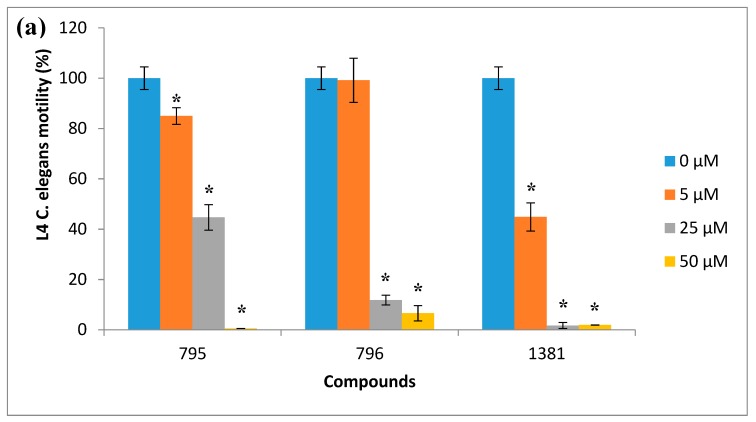

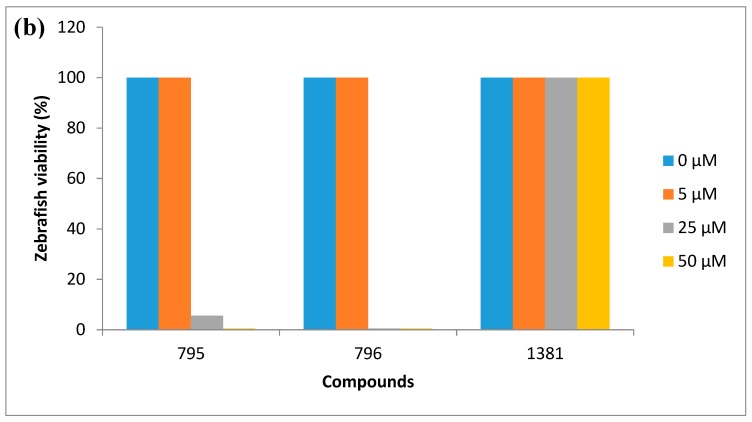
Chemolibrary compound (codes 795, 796, and 1381) effects on *C. elegans* motility and toxicity in zebrafish: (**a**) 795, 796, and 1381 were tested at 5, 25, and 50 µm against fourth-stage larvae (L4) of *C. elegans* for 18 h. The worms were incubated in K saline containing 0.015% BSA, 1% DMSO, and the compounds at different concentrations. Asterisks indicate a significant difference from the control value (* *p* ≤ 0.01 by unpaired *t*-test). Six replicates were performed per condition. (**b**) Here, 795, 796, and 1381 were tested at 5, 25, and 50 µm against 96-h postfertilization zebrafish larvae for 24 h. Eighteen zebrafish embryos per condition were incubated in E3 embryo medium at the different compound concentrations. Viability was assessed by the presence of a heartbeat under stereoscopic microscope observation after the 24-h treatment.

**Figure 6:**
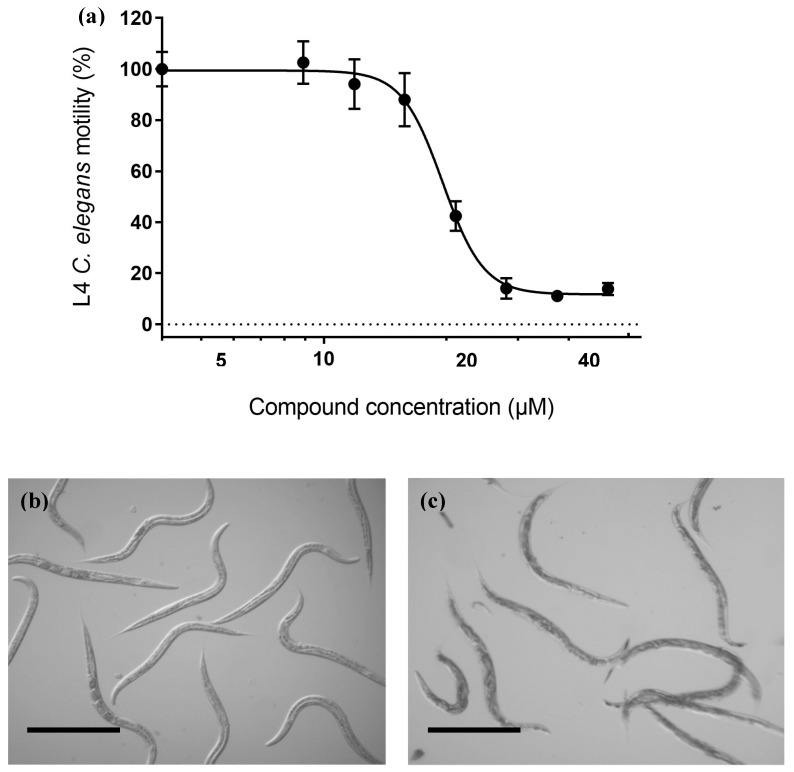
Chemolibrary compound code 1381 effects on *C. elegans* motility: (**a**) 18-h 1381 dose–response motility assay of fourth-stage larvae (L4) of *C. elegans*. The worms were incubated in K saline containing 0.015% BSA, 1% DMSO, and 1381 at different concentrations (range 8–50 µm). The EC50 was 19.6 ± 0.4 µm. (**b**,**c**) Effect of 1381 on *C. elegans*. Fourth-stage larvae (L4) *C. elegans* worms were incubated with vehicle (Panel B) or with 1381 at 50 µm (Panel C) for 18 h. Scale bar: 500 µm.

The most active compounds, 795, 796, and 1381, were then assayed at 5, 25, and 50 µm (Figure 5a), and only compound 1381 paralyzed worms at 25 and 50 µm. Although for these compounds motility did not reach zero at the highest concentration assayed, the absence of movement at 24 h was observed. Furthermore, treated L4 did not develop into adult worms. Then we examined these molecules at 5, 25, and 50 µm against zebrafish larvae as a vertebrate model (Figure 5b). Compound 1381 was active as a nematicide at 25 and 50 µm, but it did not exert any viability effect on zebrafish at 50 µm, being insoluble above this concentration. For this compound, a dose response was made, yielding an EC50 of 19.6 ± 0.4 µm (Figure 6). Additionally, we explored the toxicology profile of this compound using PROTOX toxicity prediction open software [33]. An LD50 value of 1460 mg/kg was predicted as the oral acute toxicity in a mouse, making this compound a low-toxicity class compound (Supplementary Materials Figure S1).

## 4. Discussion

In this study, we describe a whole-organism screening for nematicides using the model organism *C. elegans*. Most screens for anthelmintic discovery rely on target-based approaches. However, these approaches are most adequate for optimizing a hit. Instead, whole-organism-based screenings are powerful for identifying new hits. To this end, nematode parasites have been used, but they have limitations in large-scale screenings since they require a continuous and homogeneous source of host-derived parasites. *C. elegans* is a free-living nematode and a model organism that has recently been established as an effective cost-efficient model system for anthelmintic drug discovery [15]. The method used in that study, however, required microscopy examination of each well after five days of liquid culture. Since most known anthelmintics affect motility, we thought that WMicrotrackerTM ONE would offer a convenient method because it allows for the locomotion of *C. elegans* to be monitored and quantified automatically on microtiter plates [32]. In particular, this motility assay is nonlabor-intensive, and the readout does not require additional processing. 

We first standardized the assay and proved that it provided a clear readout with two commercial nematicides known to affect motility. Indeed, reproducible dose–response curves were obtained for levamisole and ivermectin. To fully validate the method, we screened a curated library of compounds that had already been tested for anthelmintic activity. Pathogen Box is an ideal medium-sized chemical library that had recently been tested using the nematode parasite *H. contortus* [25]. Importantly, *C. elegans* and *H. contortus* screens resulted in the identification of a single molecule present in Pathogen Box: the approved pesticide tolfenpyrad. In contrast to the method used in the study by Preston et al., which required video recording and processing [25], the method used in this study had an automatic and simple overnight readout. The results cross-validated tolfenpyrad as the single nematicide present in Pathogen Box. This highlights the importance of using publicly available curated chemical libraries for generating consistent results, and more generally for repurposing existing drugs. 

We then challenged the method with 175 compounds from our in-house chemolibrary. Interestingly, of the active molecules against eukaryotic parasites (60), 19 (32%) were active against *C. elegans*. In contrast, of the other 115 compounds selected from the library, only 9 (8%) had nematicidal activity.

**Figure 7:**
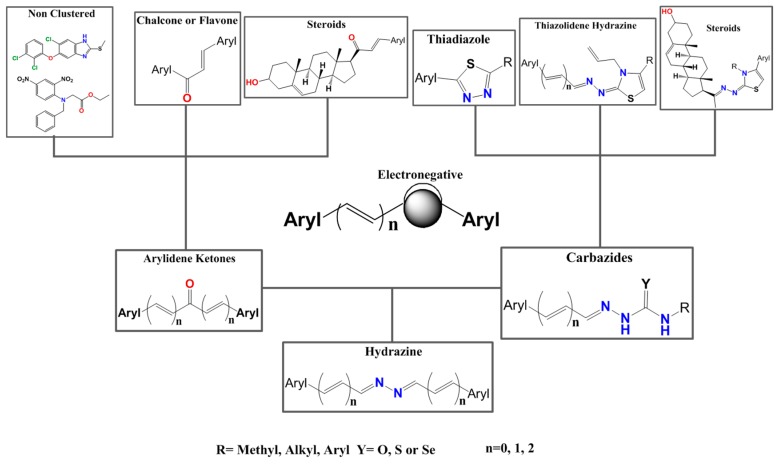
Scaffolds of active families.

Seven different compound families were active (Figure 7): arylidene ketones (8 out of the 27 compounds examined of this family), hydrazines (2 out of 4), thiazolidenehydrazines (7 out of 37), thiadiazoles (1 out of 5), carbazides (4 out of 26), steroids (2 out of 22), and chalcones (2 out of 10). Interestingly, of the 27 arylidene ketones, the most potent family against *T. cruzi* was also the most potent in this screening [26]. Inactive families in this screening were quinoxalines (7), indazoles (18), furoxanes (6), and triazines (3). Among the active molecules, we observed a repeated pattern: Conjugation of an unsaturation (one or two double bonds) with an electronegative atom in the center of the molecule (N, O, or S) and an aromatic ring (Figure 7, center). This pattern was in 26 out of the 28 active molecules, indicating that this type of structure constitutes a scaffold for future optimization. It is also important to highlight that five out of the six most potent nematicides were symmetric, with five-atom aromatic heterocycles as substituents. 

One of the most active compounds (chemolibrary code 796) is in the preclinical phase of drug development for Chagas disease, leishmaniasis, and sleeping sickness. Five out of six potent molecules (795, 796, 1140, 1245, and 1381) have never been identified as nematicides, providing new scaffolds for rational drug design with increased efficacy. Compound 1381 is particularly promising: It is effective against *C. elegans* and completely innocuous toward zebrafish, and it has a simple and low-cost synthesis. It will be relevant to test this molecule on a parasite nematode in future studies. Importantly, compounds 795, 796, 1140, and 1381 have been shown to be chemically stable in different conditions, including cell cultures [26,30,34], and metabolic studies for these compounds are currently underway.

The advantages of *C. elegans* as a model organism have not been fully exploited in nematicide discovery. We validated an extremely simple and reliable HTS method using locomotion as a readout to search for nematicides, taking advantage of the free-living model organism *C. elegans*. 

## Figures and Tables

**Table 1:** The 28 selected active molecules according to the cut-off criteria (mild to moderate activity: % of remaining motility 25%–65% at 20-50 µm; potent activity: % of remaining motility <25% at 20–50 µm).

Chemolibrary Code	Structure	Motility (%) at 25–50 µm
**796**	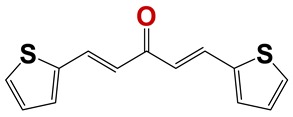	7
**694**	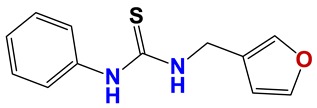	64
**795**	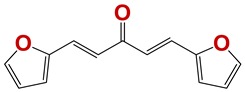	0
**808**	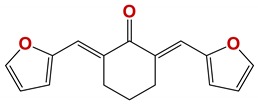	53
**804**	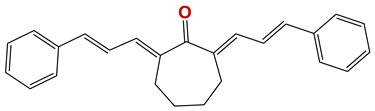	63
**798**	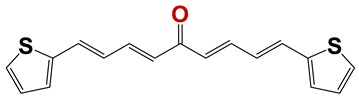	63
**809**	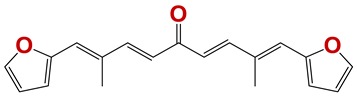	55
**1282**	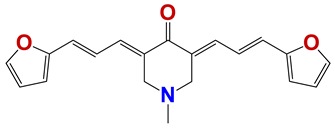	62
**1245**	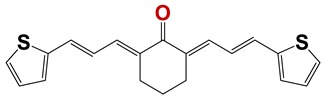	23
**262**	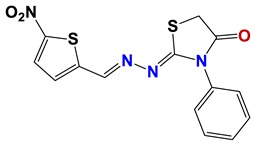	59
**145**	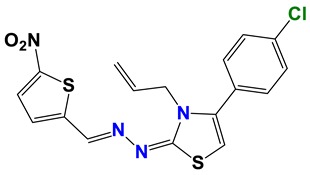	60
**313**	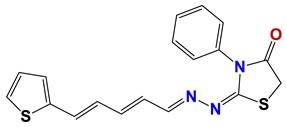	63
**314**	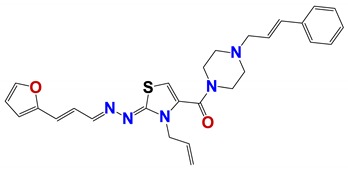	60
**1364**	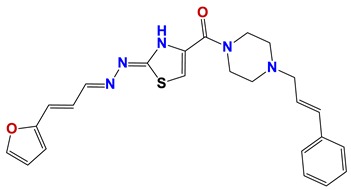	61
**1381**	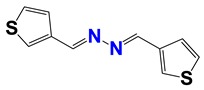	2
**1140**	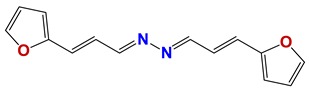	23
**782**	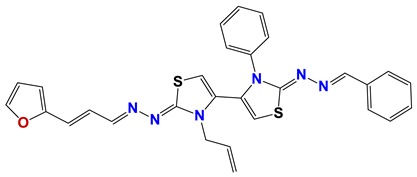	61
**724**	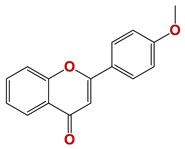	63
**731**	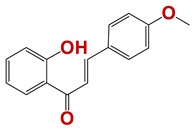	8
**813**	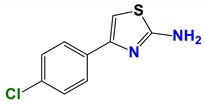	50
**1384**	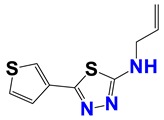	55
**568**	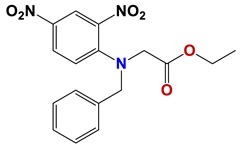	58
**1367**	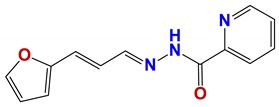	63
**1219**	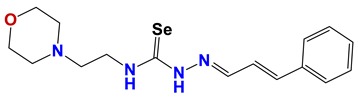	62
**1377**	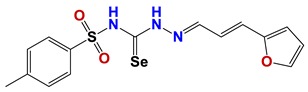	45
**1278**	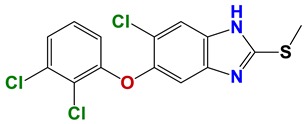	60
**1287**	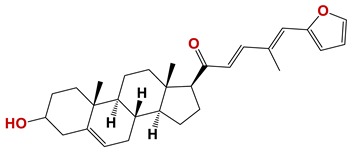	61
**1272**	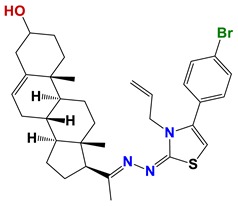	55

## Supplementary Materials

The following are available online at https://www.mdpi.com/2306-7381/6/1/29/s1, Figure S1: Toxicology profile of compound 1381 using PROTOX toxicity prediction open software, Table S1: List of the 175 selected molecules from the in house library (LIDENSA Chemolibrary) examined in this work and their activity on *C. elegans* motility.
