# Supplementary material for: Caenorhabditis elegans Infrared-Based Motility Assay Identified New Hits for Nematicide Drug Development
Source: Vet Sci. 2019 Mar 17;6(1):29. doi: 10.3390/vetsci6010029 (PMC6466232; doi:10.3390/vetsci6010029)
Supplement: Supplementary file 1 [file vetsci-06-00029-s001.pdf]

Oral toxicity prediction results for input compound

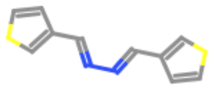

Predicted LD50: 1460mg/kg

Predicted Toxicity Class: 4

1	2	3	4	5	6

Average similarity: 37.99%

Prediction accuracy: 23%

Name	C1(/C=N/N=C/C2=CSC=
Molweight	220.31
Number of hydrogen bond acceptors	2
Number of hydrogen bond donors	0
Number of atoms	14
Number of bonds	15
Number of rings	2

Toxicity Model Report

Classification	Target	Shorthand	Prediction	Probability
Organ toxicity	Hepatotoxicity	dili	Inactive	0.53
Toxicity end points	Carcinogenicity	carcino	Active	0.58
Toxicity end points	Immunotoxicity	immuno	Inactive	0.99
Toxicity end points	Mutagenicity	mutagen	Inactive	0.54
Toxicity end points	Cytotoxicity	cyto	Inactive	0.80
Tox21-Nuclear receptor signalling pathways	Aryl hydrocarbon Receptor (AhR)	nr_ahr	Inactive	0.57
Tox21-Nuclear receptor signalling pathways	Androgen Receptor (AR)	nr_ar	Inactive	0.82
Tox21-Nuclear receptor signalling pathways	Androgen Receptor Ligand Binding Domain (AR-LBD)	nr_ar_lbd	Inactive	0.8
Tox21-Nuclear receptor signalling pathways	Aromatase	nr_aromatase	Inactive	0.94
Tox21-Nuclear receptor signalling pathways	Estrogen Receptor Alpha (ER)	nr_er	Inactive	0.54
Tox21-Nuclear receptor signalling pathways	Estrogen Receptor Ligand Binding Domain (ER-LBD)	nr_er_lbd	Inactive	0.97
Tox21-Nuclear receptor signalling pathways	Peroxisome Proliferator Activated Receptor Gamma (PPAR-Gamma)	nr_ppar_gamma	Inactive	0.77
Tox21-Stress response pathways	Nuclear factor (erythroid-derived 2)-like 2/antioxidant responsive element (nrf2/ARE)	sr_are	Inactive	0.68
Tox21-Stress response pathways	Heat shock factor response element (HSE)	sr_hse	Inactive	0.68
Tox21-Stress response pathways	Mitochondrial Membrane Potential (MMP)	sr_mmp	Inactive	0.86
Tox21-Stress response pathways	Phosphoprotein (Tumor Supressor) p53	sr_p53	Inactive	0.73
Tox21-Stress response pathways	ATPase family AAA domain-containing protein 5 (ATAD5)	sr_atad5	Inactive	0.74

Table S1. List of the 175 selected molecules from the in house library (LIDENSA Chemolibrary) examined in this work and their activity on *C. elegans* motility. The compound code number, structural formula, concentration used and remaining motility are shown. Color code: yellow for moderate activity (65-25% of motility), green for potent activity (25-0% of motility).

Chemolibrary code	Structure	Concentration (μM)	Motility (%)
**1285**	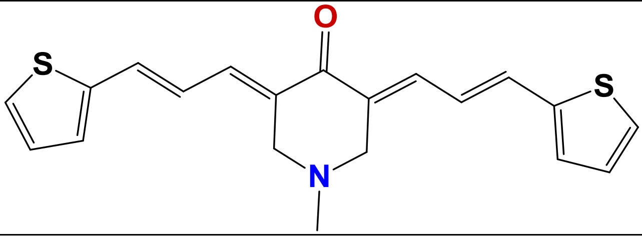	24	72
**1311**	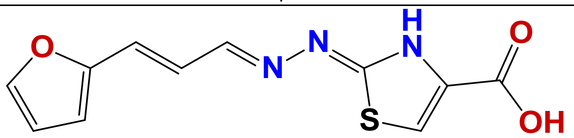	24	68
**1319**	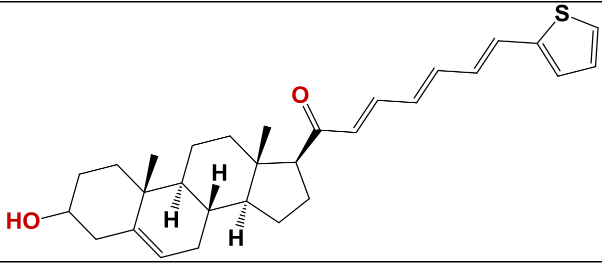	18	100
**1257**	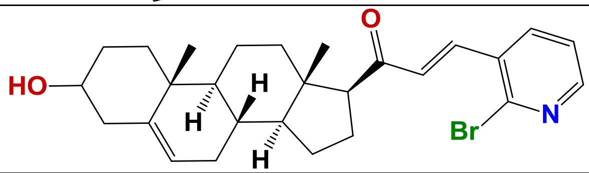	21	108
**1282**	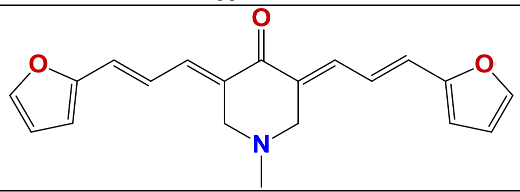	28	62
**1289**	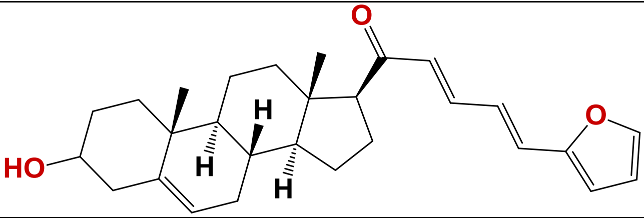	17	99
**1097**	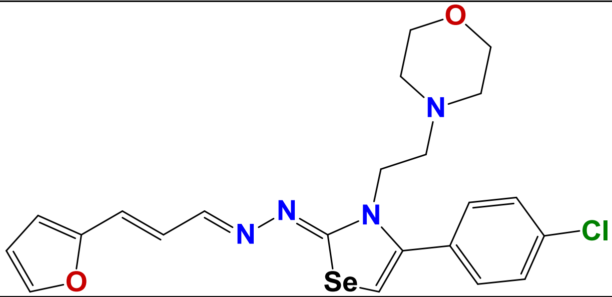	27	87
**1366**	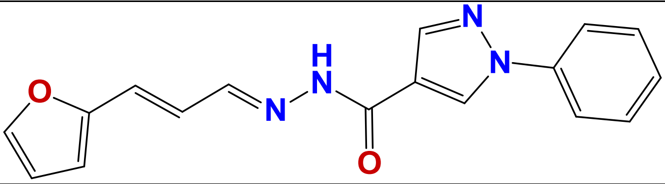	29	111

1258	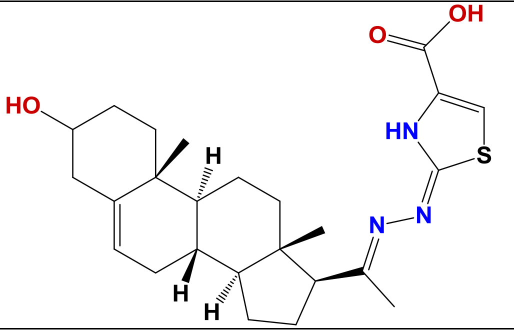	27	116
1219	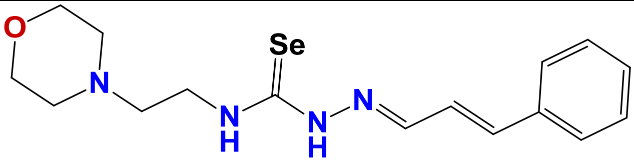	40	62
1312	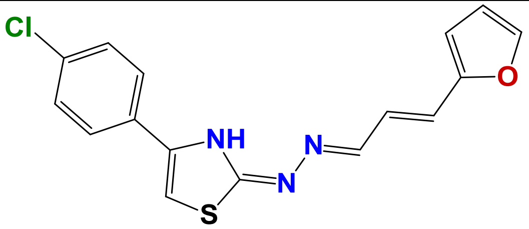	50	94
1368	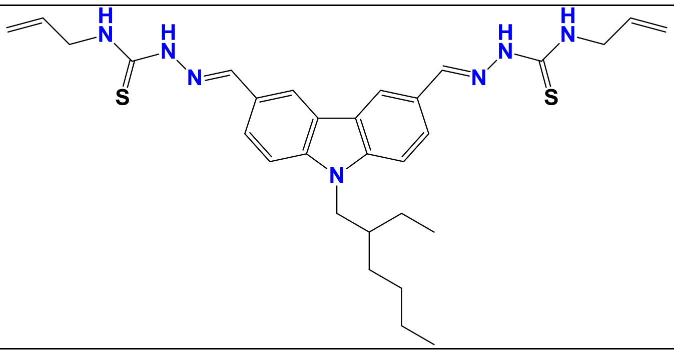	19	105
1287	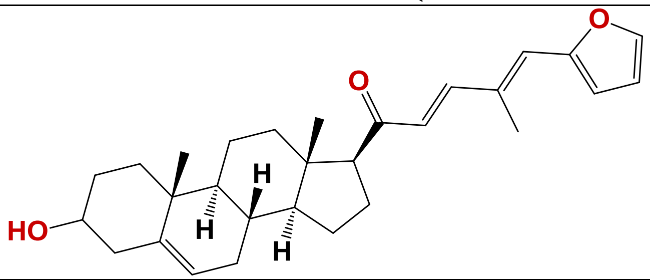	50	61
1367	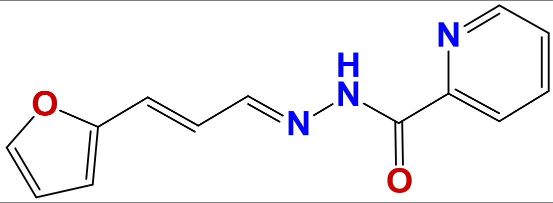	50	63
1316	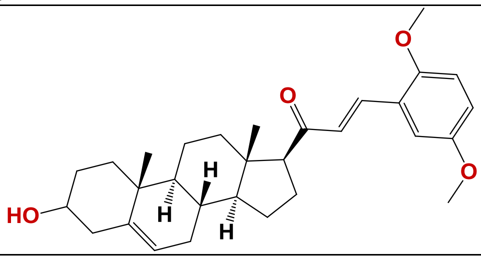	21	106

1140	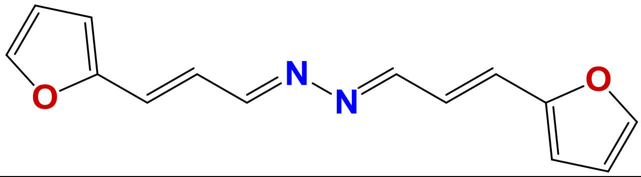	50	23
1291	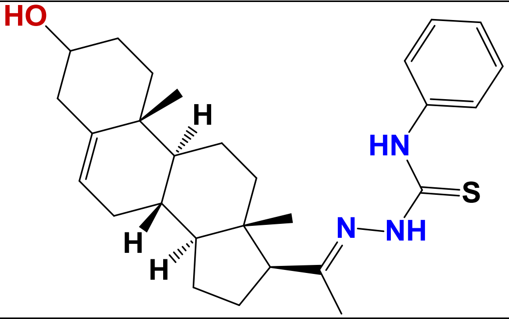	20	216
1262	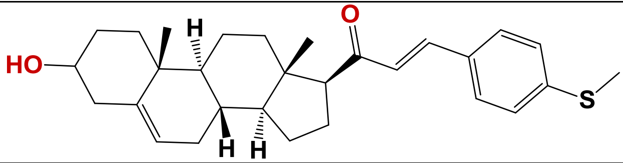	17	200
1154	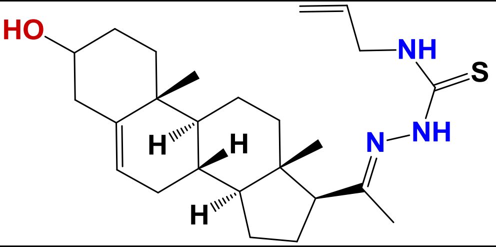	43	107
1279	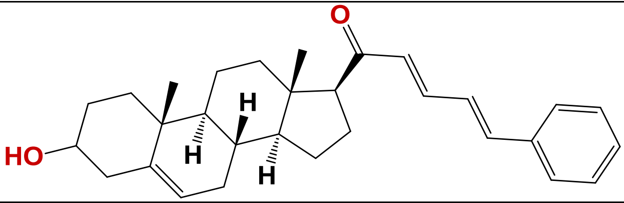	20	109
1307	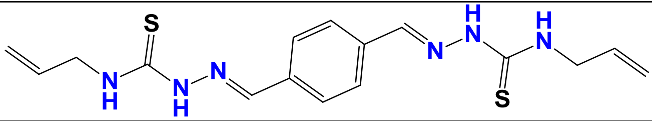	14	107
1369	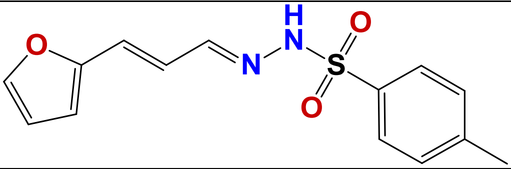	37	84
1288	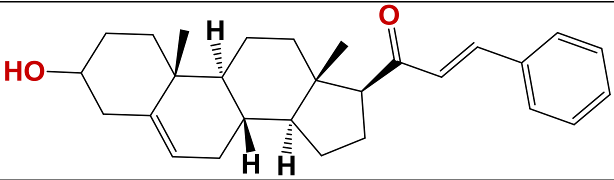	18	126
1290	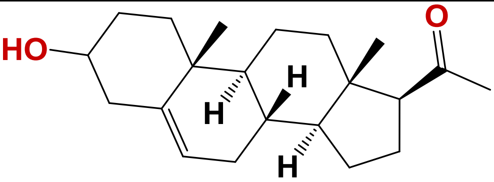	19	148

**1222**	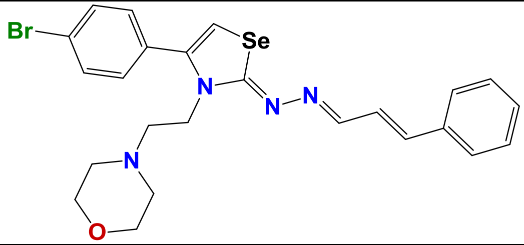	13	145
**1364**	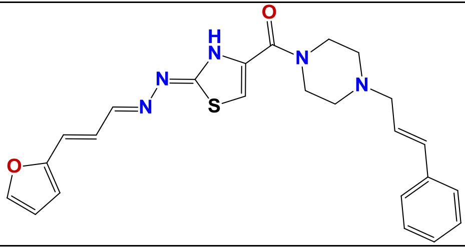	29	61
**1310**	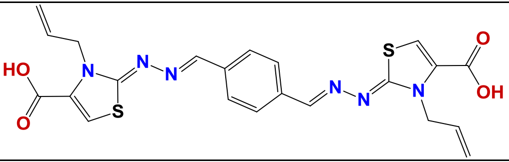	30	115
**1281**	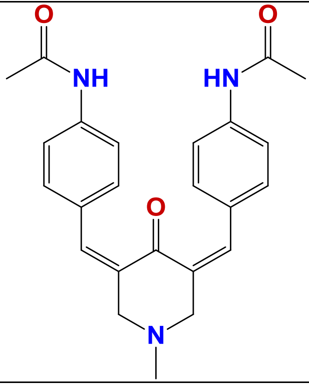	20	122
**1263**	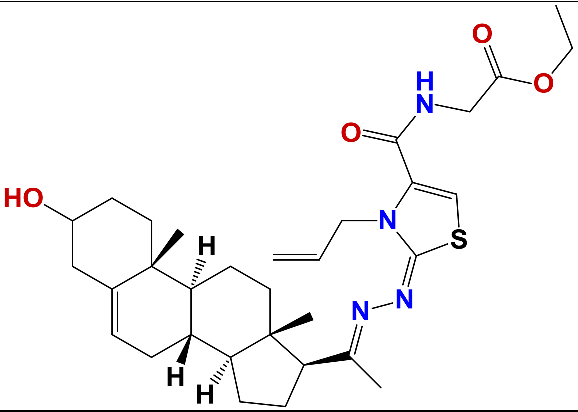	18	120
**1261**	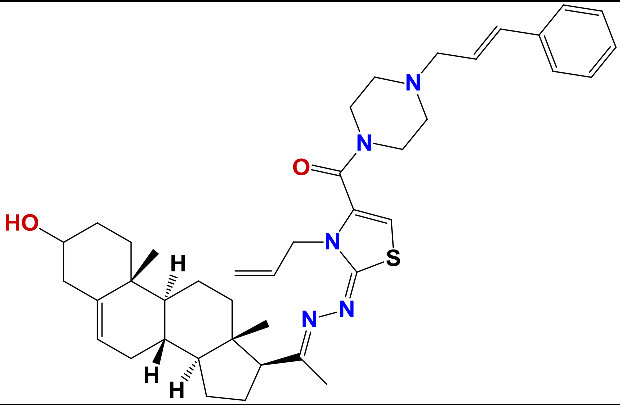	19	119

**1145**	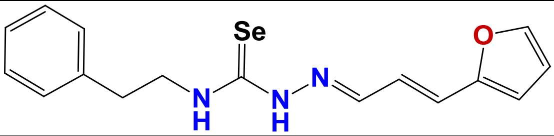	19	139
**1260**	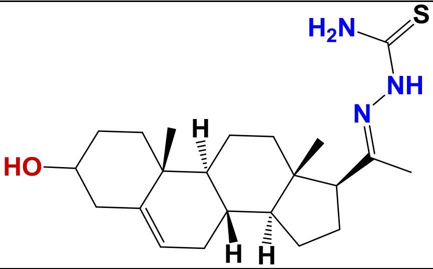	27	161
**1144**	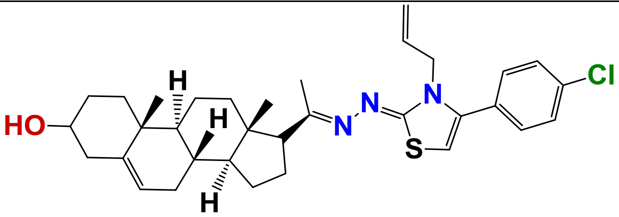	30	86
**1317**	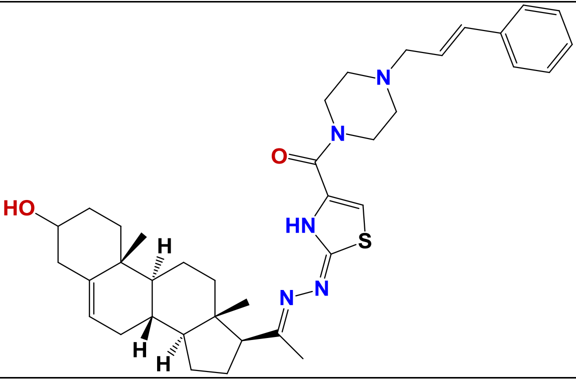	27	142
**1365**	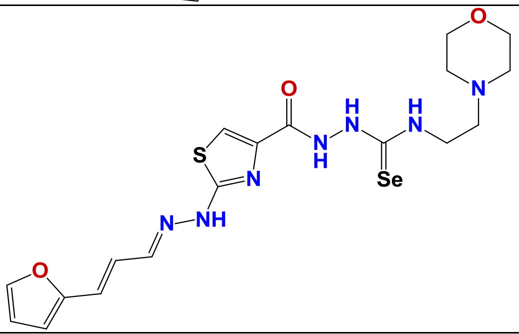	33	105
**1284**	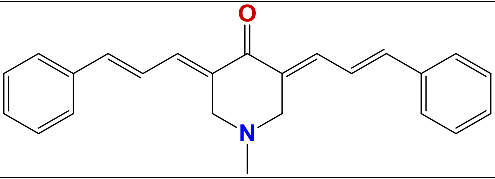	38	98

1272	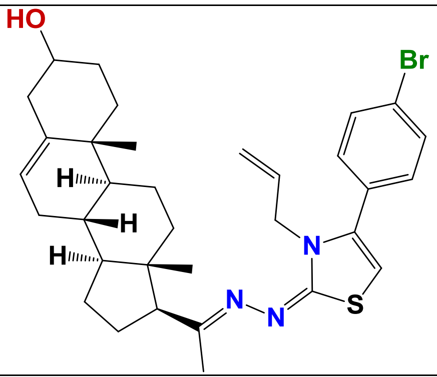	25	55
1256	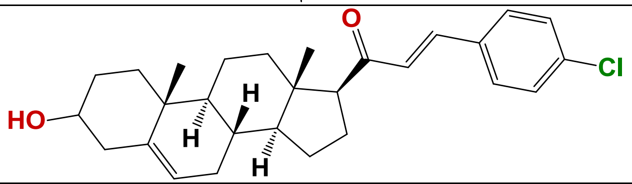	23	105
1259	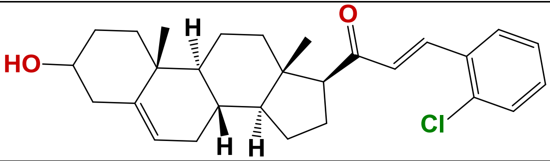	35	110
1087	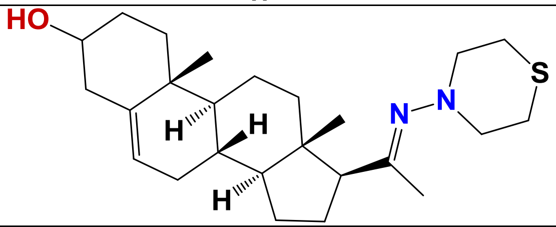	20	112
1125	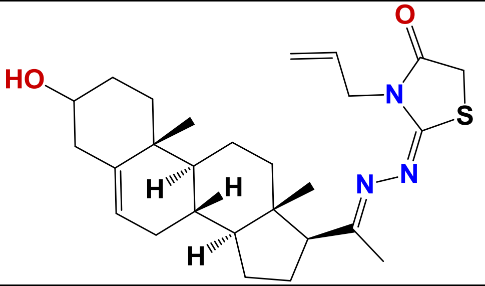	23	134
1286	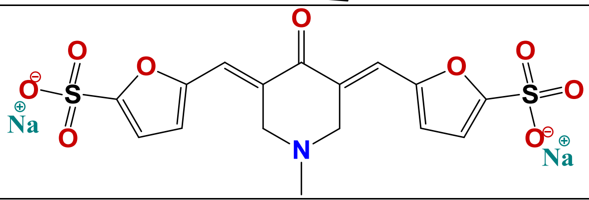	17	139
463	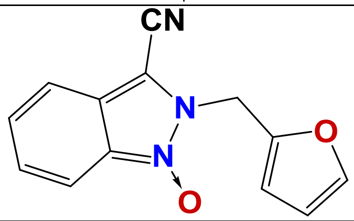	44	173
458	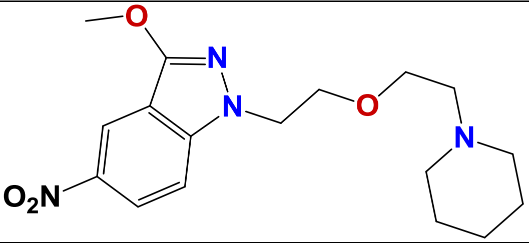	17	142

**198**	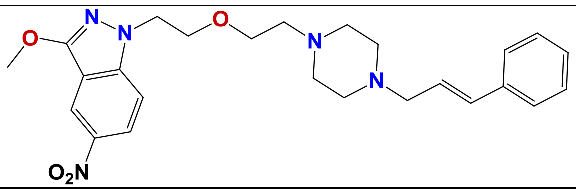	15	120
**505**	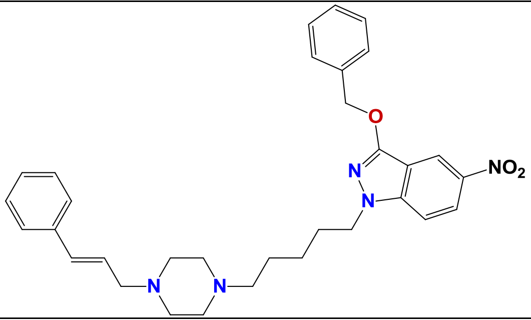	50	174
**1293**	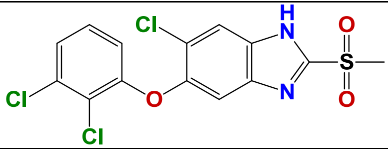	18	146
**286**	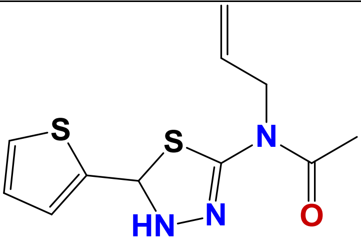	28	107
**1318**	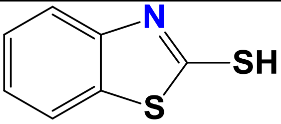	30	104
**791**	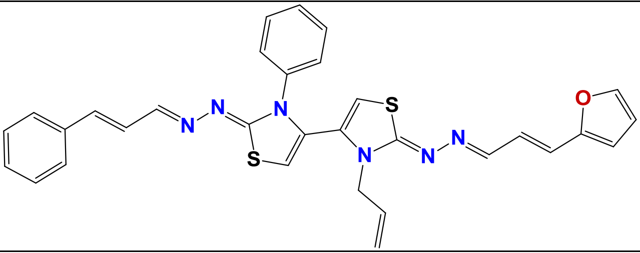	50	116
**523**	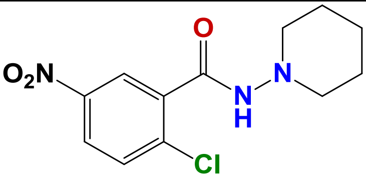	28	92
**221**	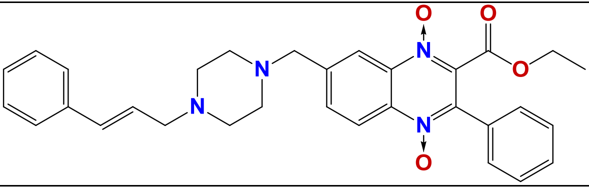	15	185
**1184**	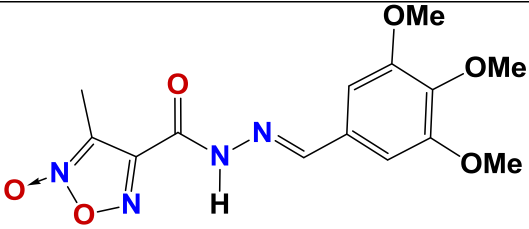	18	127

**292**	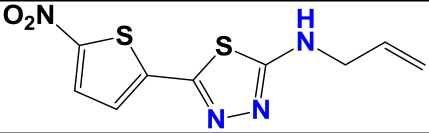	14	85
**691**	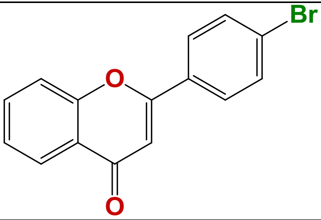	24	76
**658**	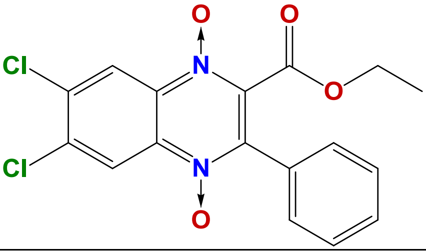	14	111
**297**	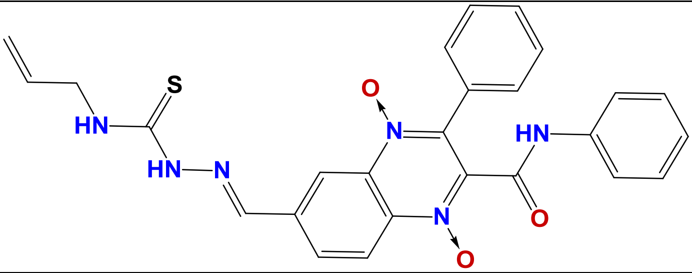	50	178
**421**	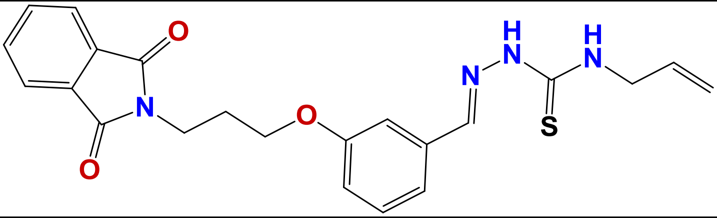	20	107
**423**	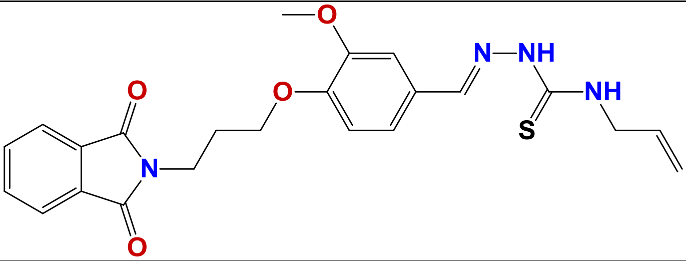	18	106
**519**	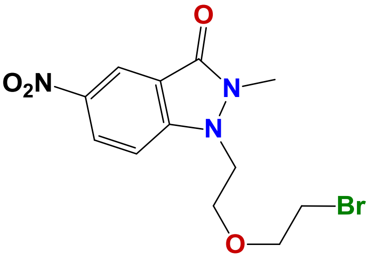	45	106
**486**	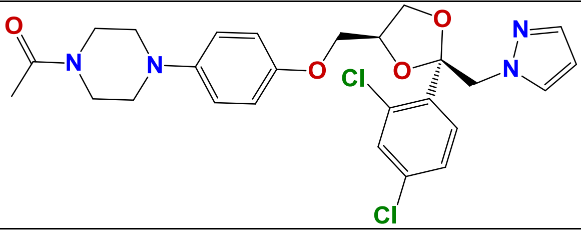	13	99

**520**	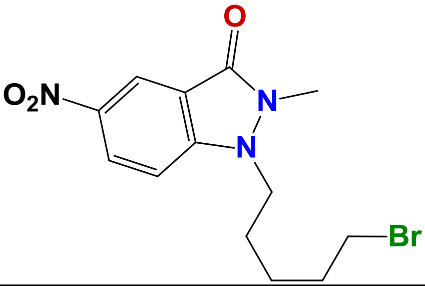	40	87
**915**	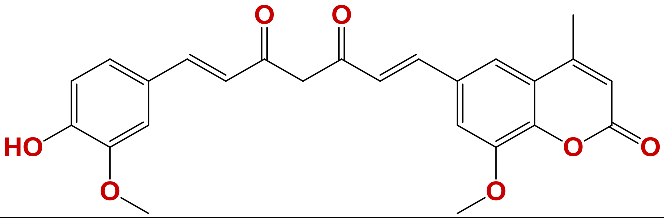	50	187
**1099**	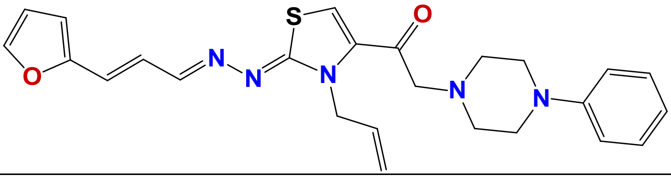	50	80
**734**	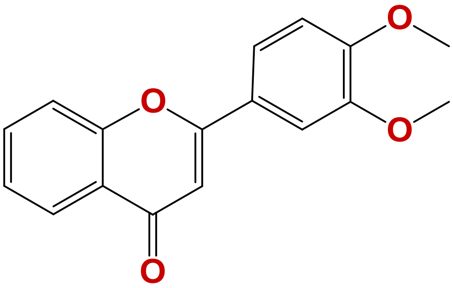	21	119
**882**	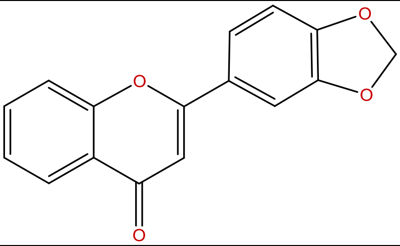	42	137
**1134**	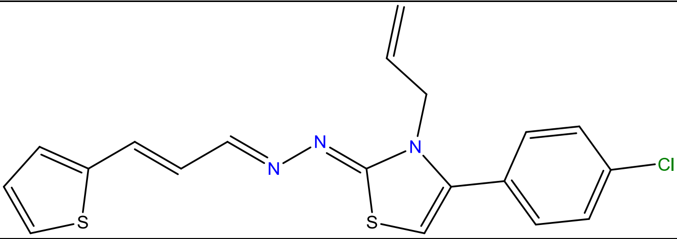	13	112
**1170**	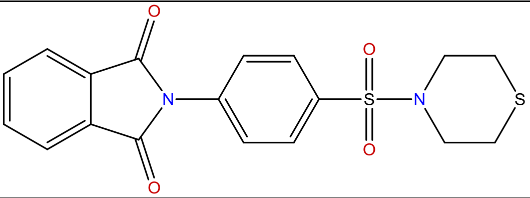	24	110

**360**	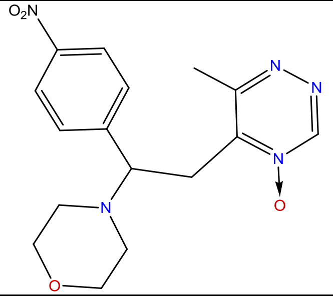	50	101
**1098**	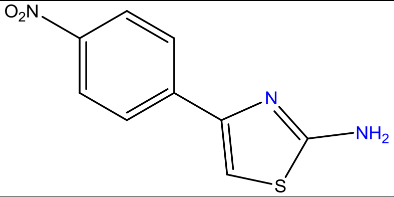	50	188
**1342**	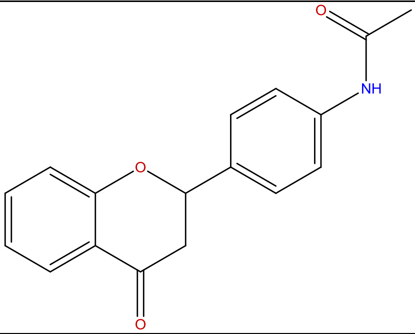	20	193
**210**	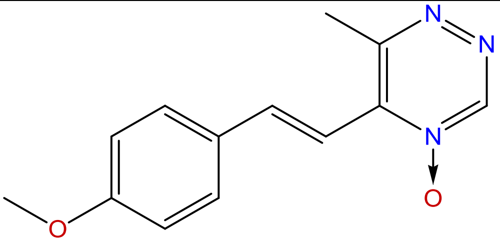	17	135
**1204**	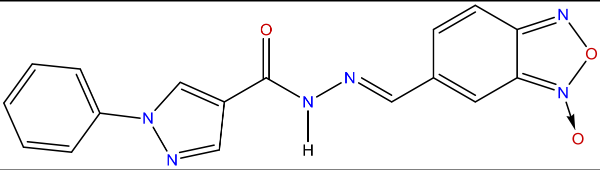	22	156
**1308**	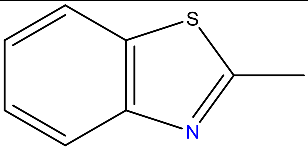	50	113
**287**	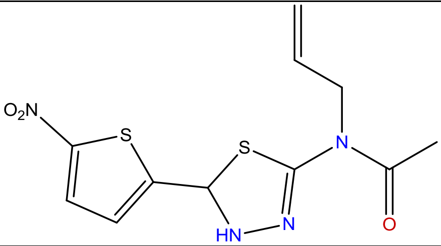	28	75

**284**	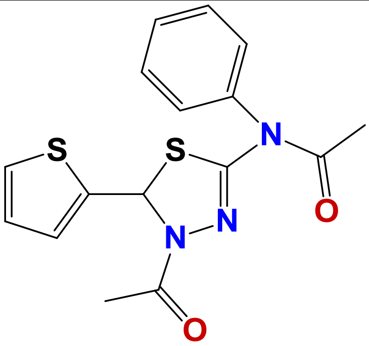	25	102
**1218**	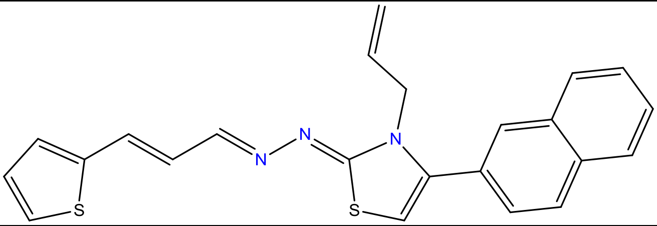	23	107
**1199**	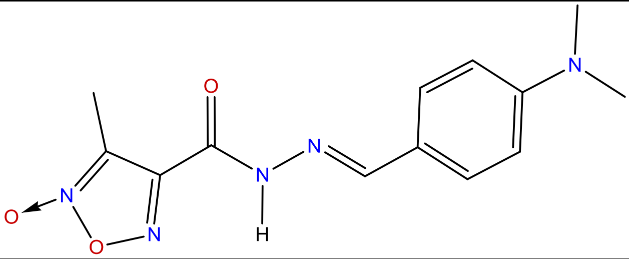	50	116
**503**	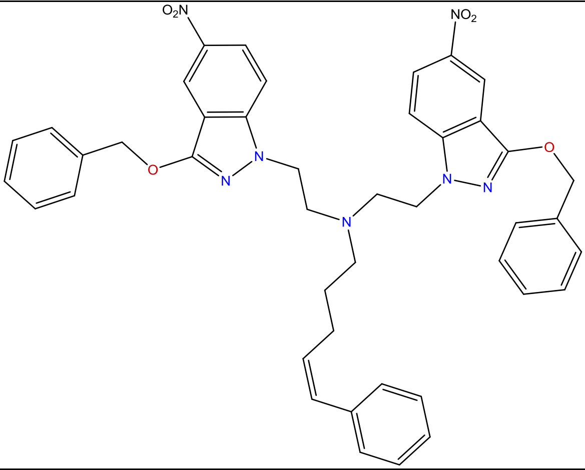	50	70
**1122**	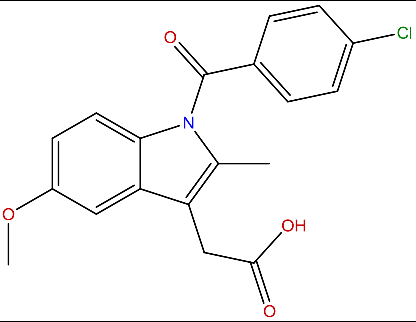	15	99
**1183**	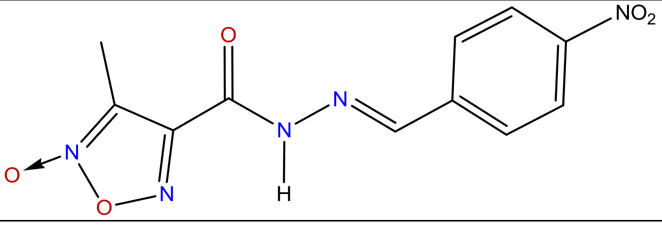	39	194

**1206**	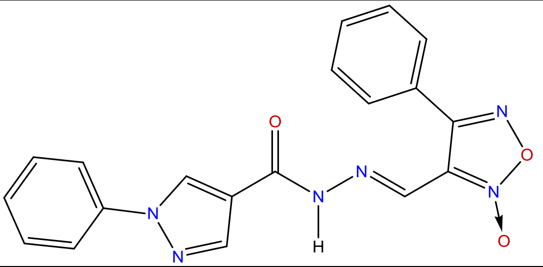	50	101
**1187**	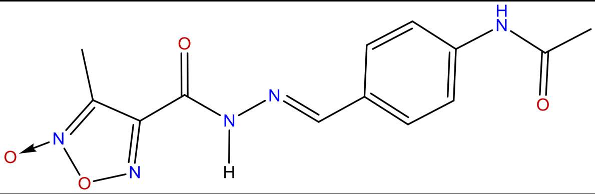	44	89
**150**	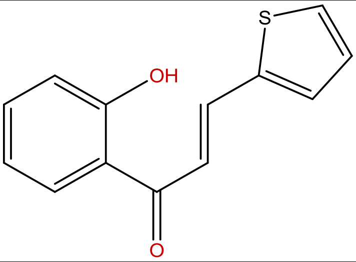	37	78
**1121**	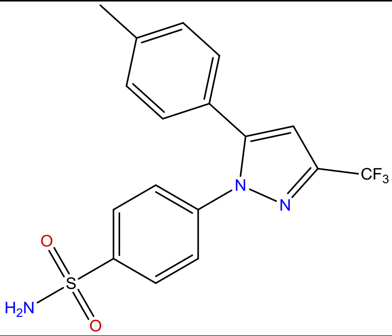	38	69
**879**	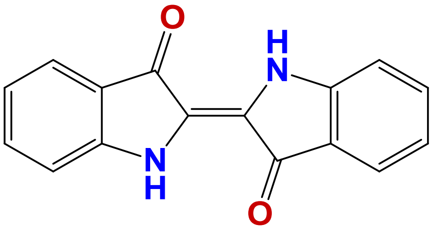	35	67
**385**	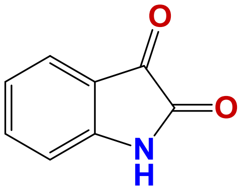	50	83

**1234**	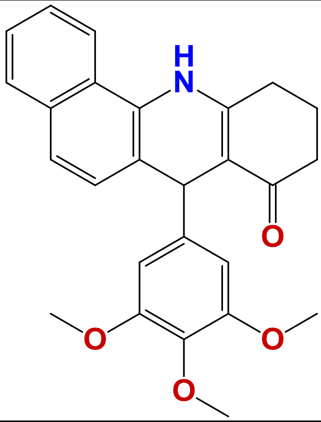	13	80
**270**	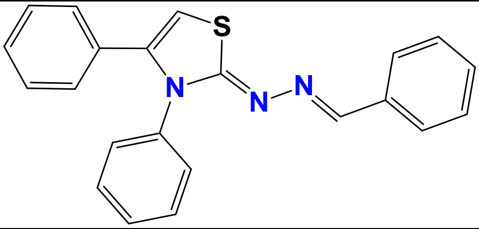	33	78
**63**	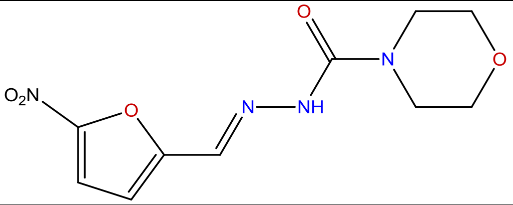	50	80
**137**	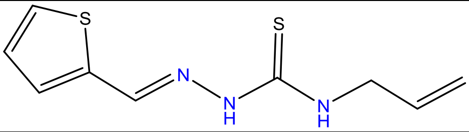	50	98
**240**	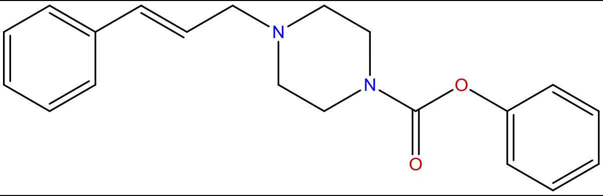	50	128
**568**	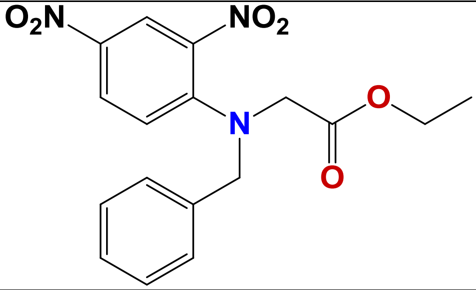	50	58
**940**	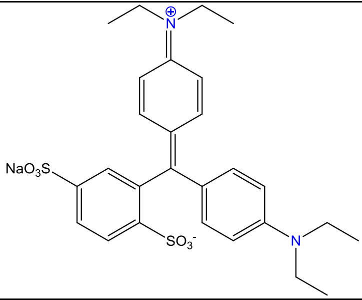	50	102

**731**	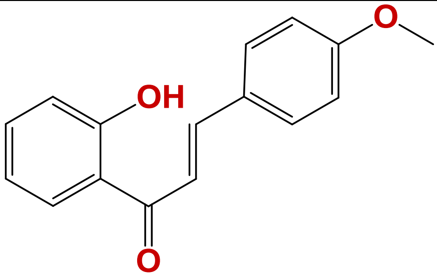	50	8
**124**	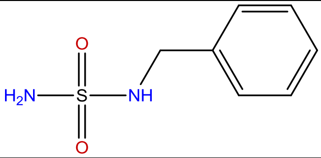	50	95
**884**	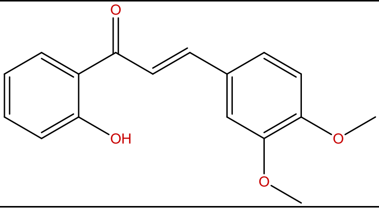	50	74
**724**	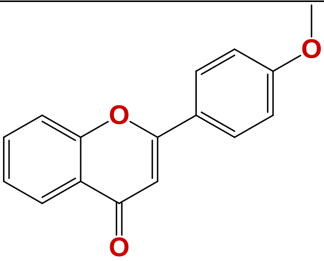	50	63
**990**	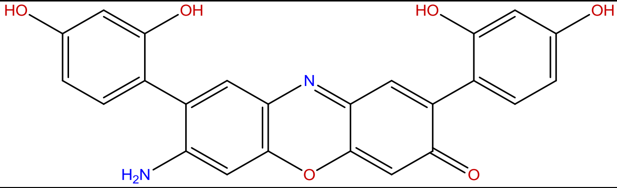	50	86
**151**	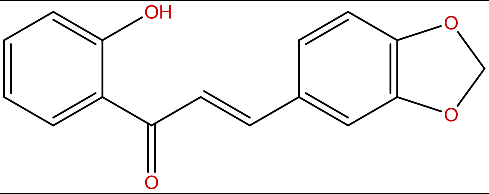	50	83
**813**	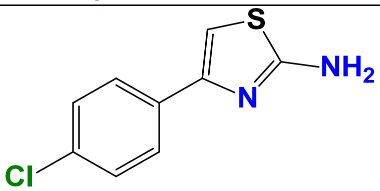	50	50
**1278**	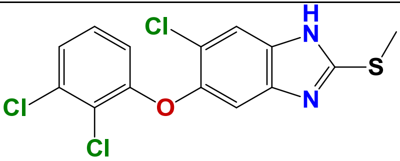	50	60
**810**	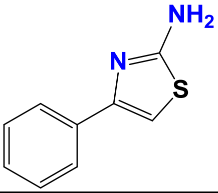	50	93

**1337**	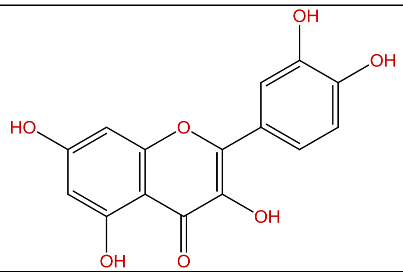	50	101
**139**	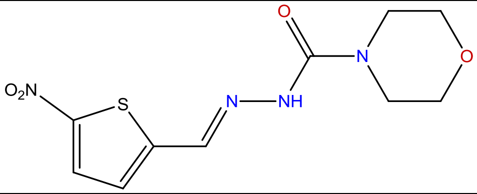	50	120
**608**	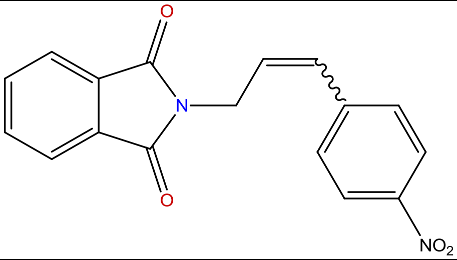	39	68
**706**	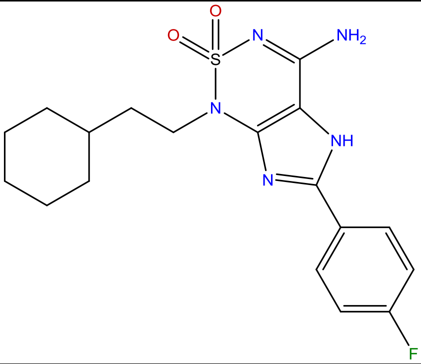	50	79
**457**	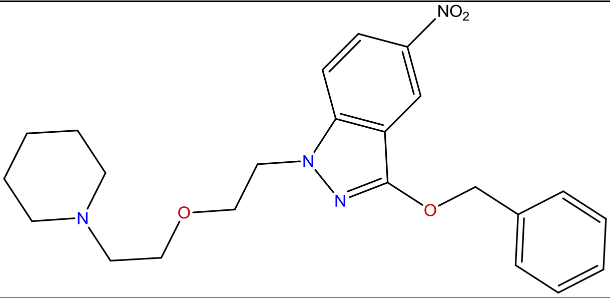	38	87
**1129**	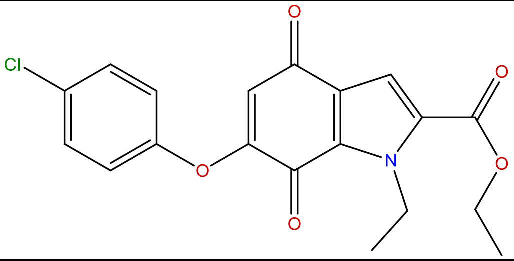	50	87

**1336**	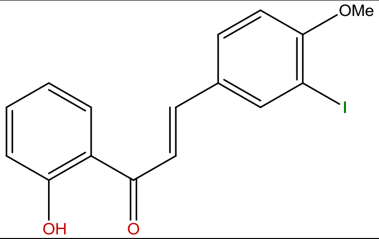	50	113
**485**	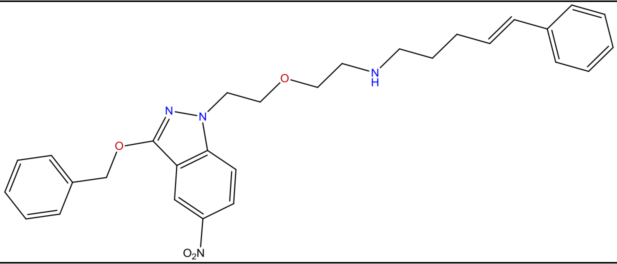	50	76
**313**	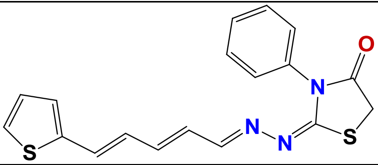	44	63
**1103**	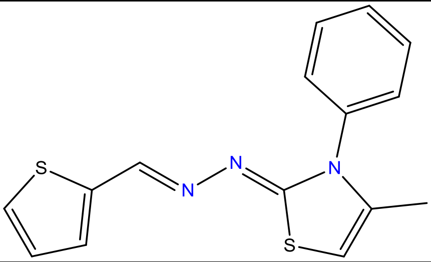	36	68
**782**	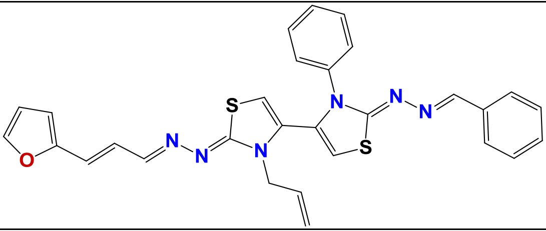	50	61
**1174**	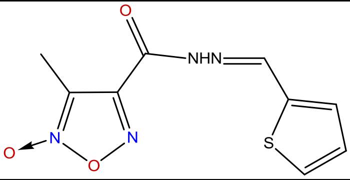	50	105
**910**	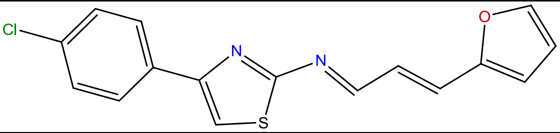	50	84
**784**	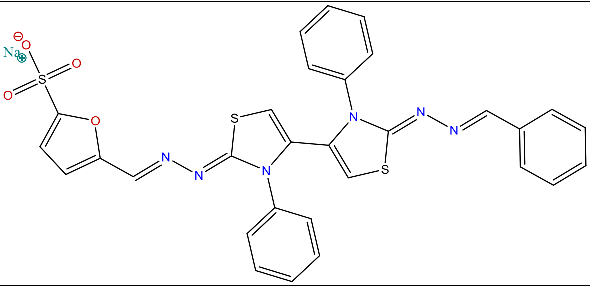	50	102

**489**	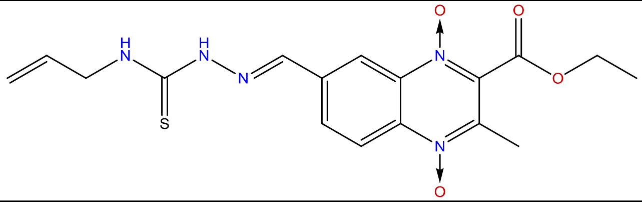	45	85
**144**	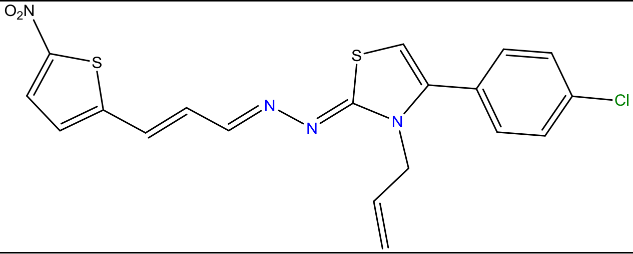	50	89
**310**	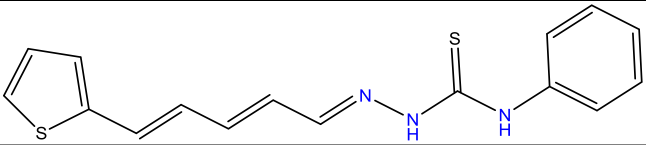	50	169
**272**	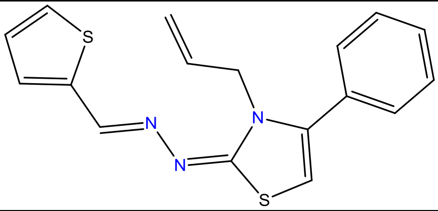	50	71
**145**	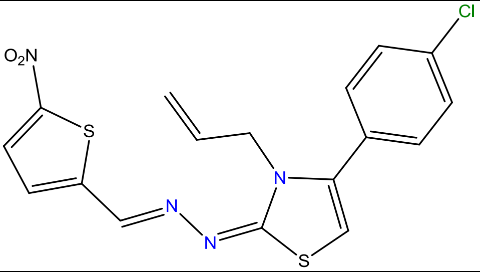	50	60
**783**	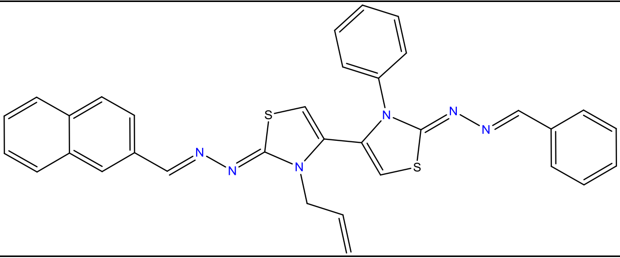	50	83
**301**	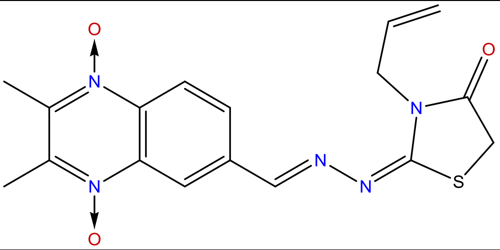	36	94
**901**	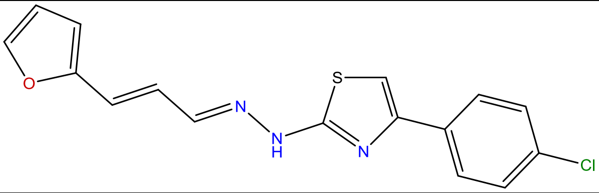	50	87

**194**	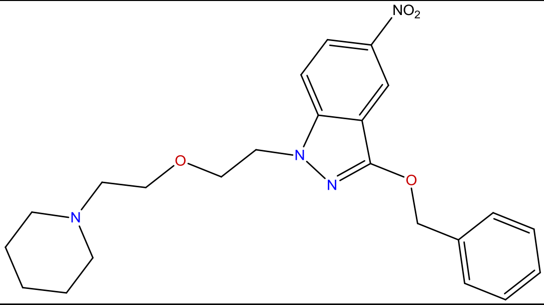	50	78
**273**	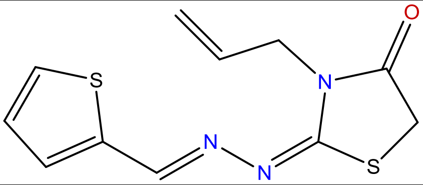	50	93
**293**	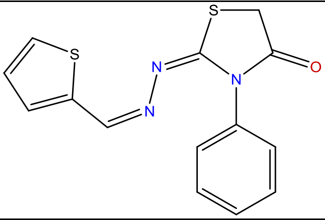	50	111
**262**	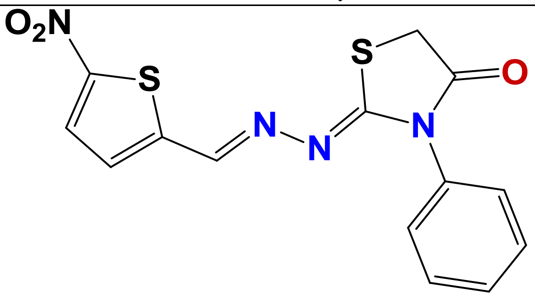	47	59
**129**	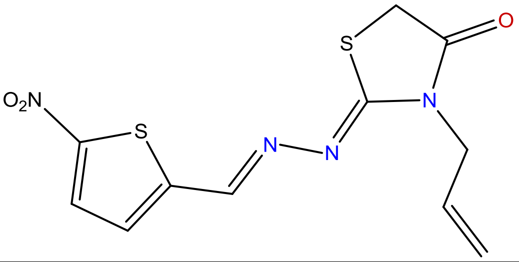	50	105
**288**	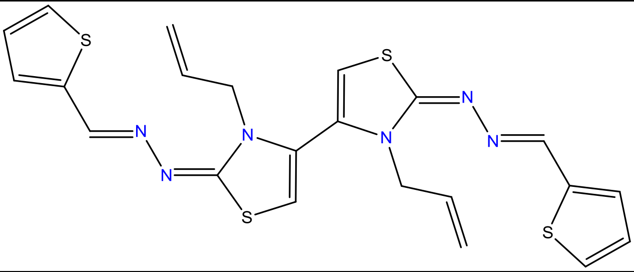	50	110
**199**	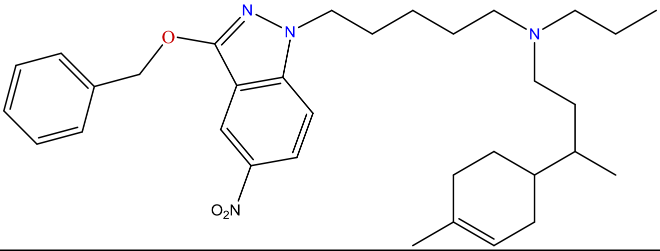	50	104

**282**	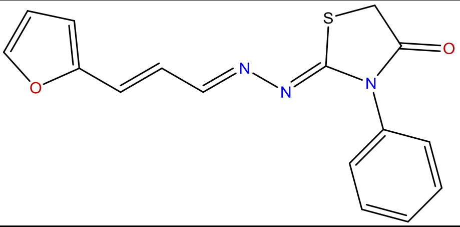	50	75
**214**	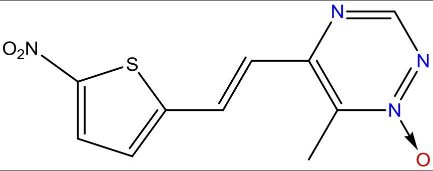	50	94
**279**	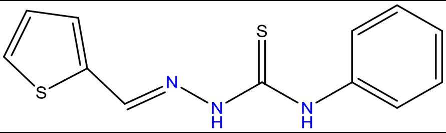	50	89
**1147**	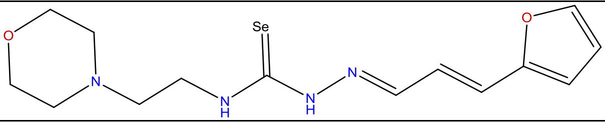	50	90
**1383**	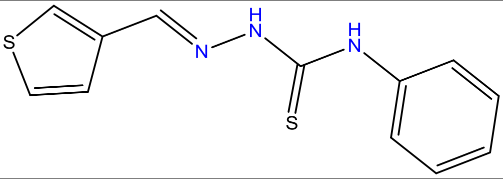	50	90
**694**	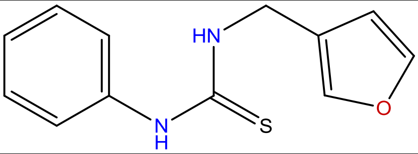	50	64
**1381**	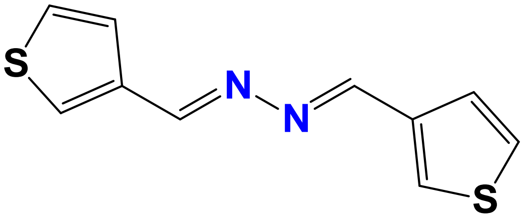	50	2
**1379**	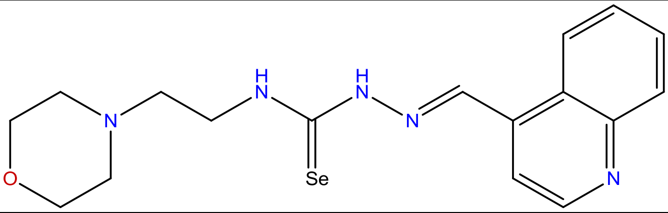	50	100
**1380**	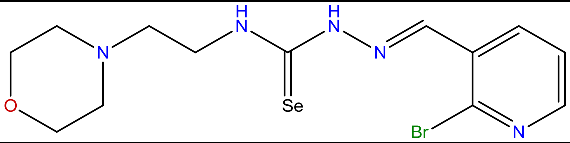	50	132
**911**	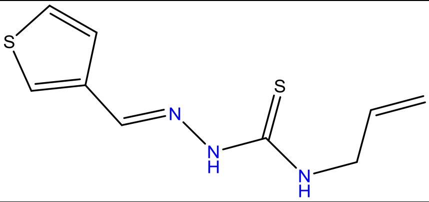	50	168

**1377**	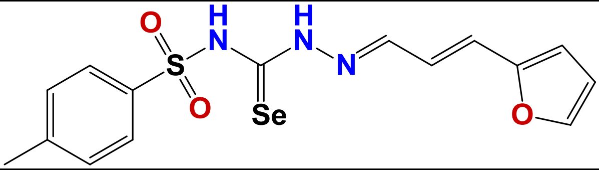	50	45
**1378**	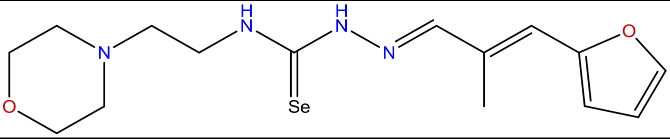	50	84
**1385**	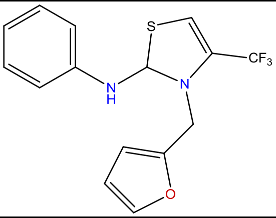	50	85
**1384**	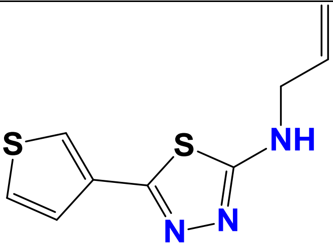	50	55
**872**	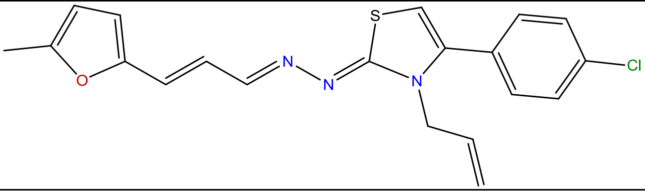	50	82
**796**	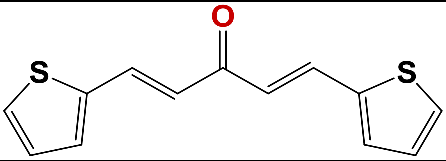	50	7
**1019**	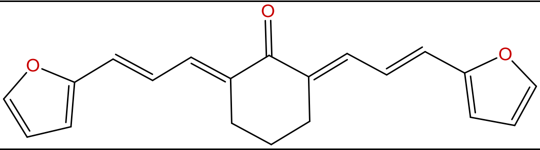	50	74
**1018**	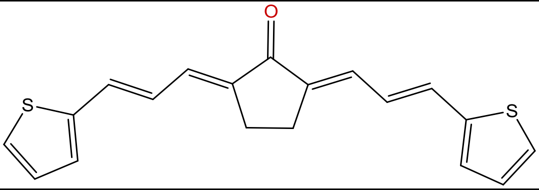	34	115

**314**	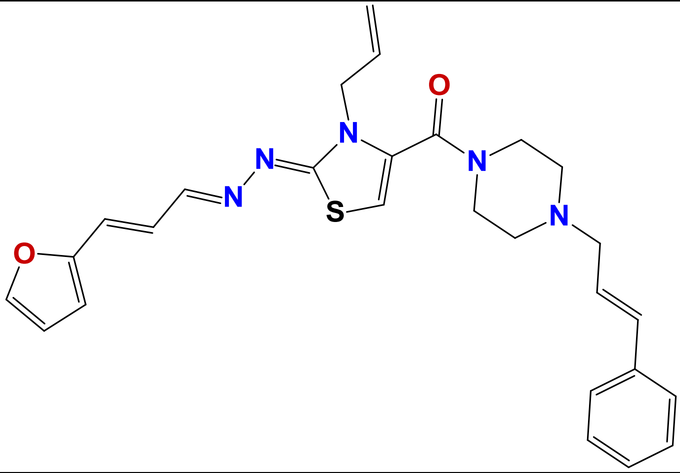	45	60
**877**	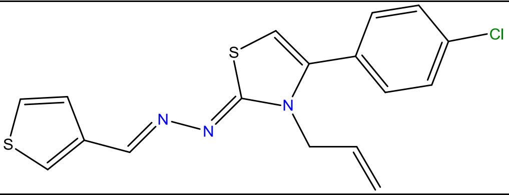	50	68
**912**	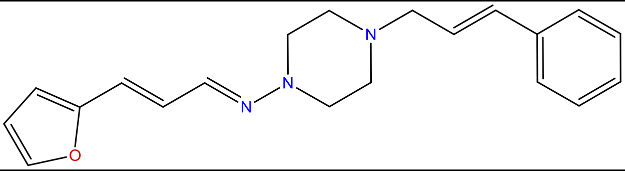	50	92
**873**	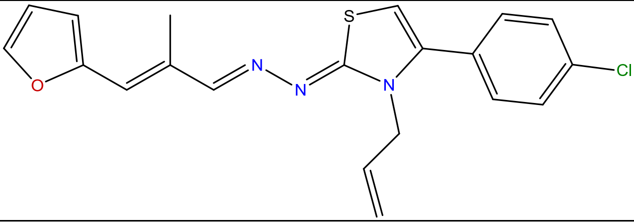	50	67
**809**	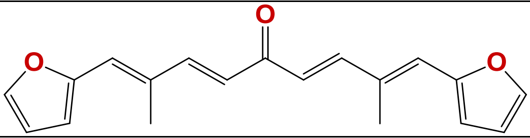	50	55
**1223**	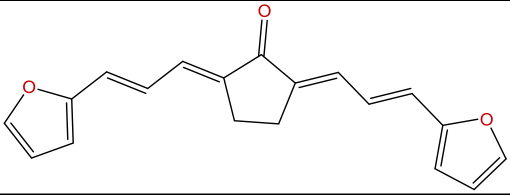	50	96
**798**	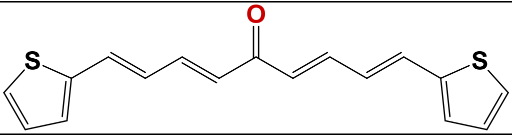	39	63
**266**	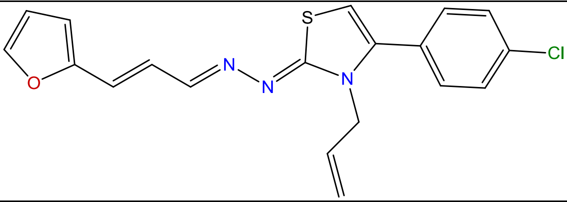	36	124

**1245**	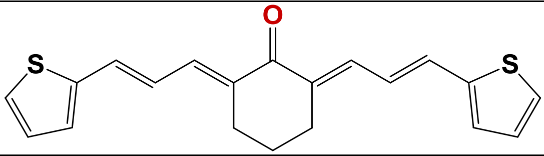	50	23
**793**	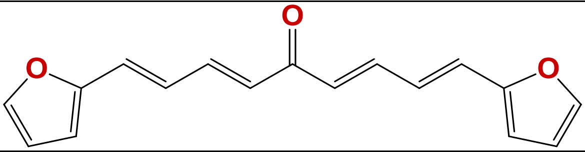	50	68
**795**	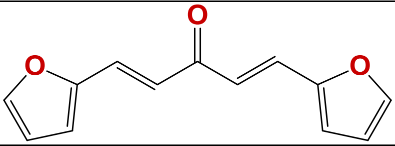	50	0
**1102**	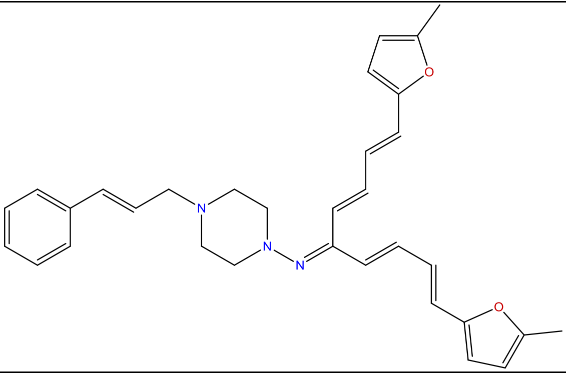	50	114
**808**	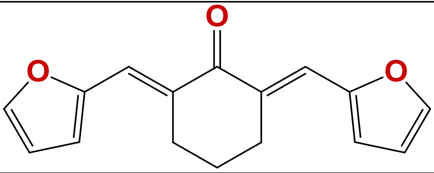	50	53
**1248**	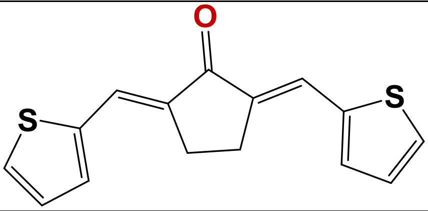	50	64
**807**	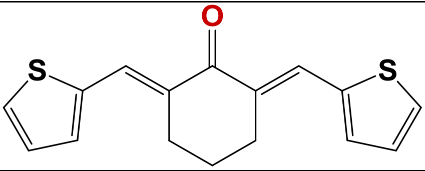	50	82
**797**	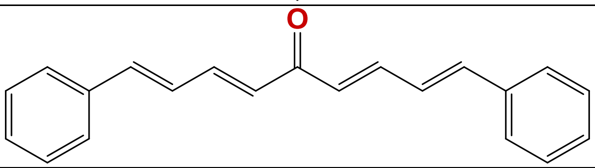	50	82
**1247**	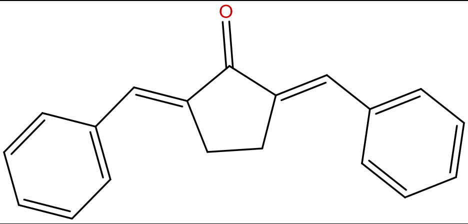	50	80

**1246**	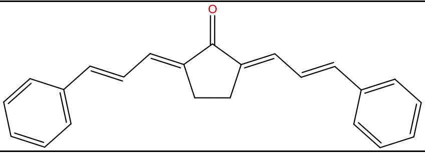	50	86
**799**	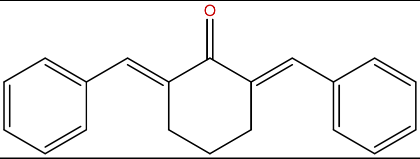	50	83
**800**	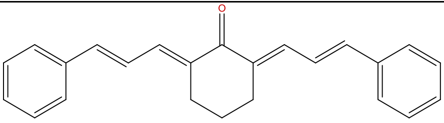	50	73
**803**	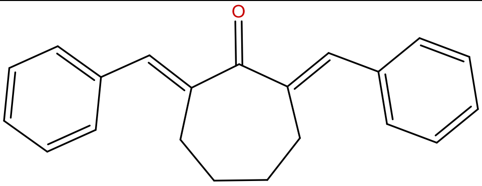	50	78
**804**	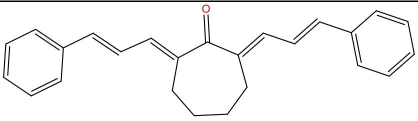	50	63
**295**	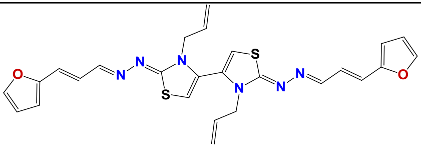	50	86
**1387**	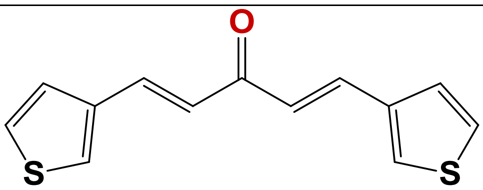	50	69
**1414**	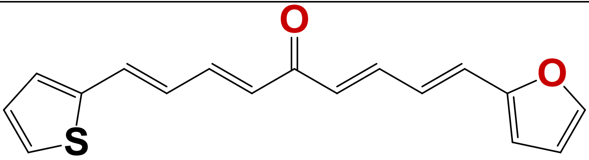	50	73
